# Minimal changes in microbial abundances and diversity over 7 years of emplacement for modules of compacted bentonite exposed to natural groundwater

**DOI:** 10.1128/aem.01950-24

**Published:** 2025-02-25

**Authors:** Harmanpreet S. Sidhu, Katja Engel, Sian E. Ford, Peter Keech, Mehran Behazin, W. Jeffrey Binns, Nivetha Srikanthan, Myrna J. Simpson, Josh D. Neufeld, Gregory F. Slater

**Affiliations:** 1 School of Earth, Environment & Society, McMaster University 152956 , Hamilton, Ontario, Canada; 2 Department of Biology, University of Waterloo 98689 , Waterloo, Ontario, Canada; 3 Nuclear Waste Management Organization 278230 , Toronto, Ontario, Canada; 4 Department of Physical & Environmental Sciences, University of Toronto Scarborough 686144 , Toronto, Ontario, Canada; University of Delaware, Lewes, Delaware, USA

**Keywords:** geomicrobiology, deep biosphere, subsurface microbiology, deep geological repository

## Abstract

The multi-national *in situ* Materials Corrosion Test (MaCoTe), being conducted at the Grimsel Test Site in Switzerland, assesses the stability of bentonite as it may be utilized within deep geological repositories (DGRs), which are proposed for the safe, long-term disposal of used nuclear fuel. This experiment provides an opportunity for long-term assessments of changes in microbial communities associated with compacted subsurface bentonite samples exposed to a natural groundwater. Leveraging samples from MaCoTe, herein, we report temporal data for the abundance and community composition of microorganisms associated with compacted bentonite samples emplaced over 7 years under *in situ* subsurface conditions. Phospholipid fatty acid analysis, 16S rRNA gene quantification and sequencing, cultivation, and natural organic matter analyses all indicated no significant changes for microbial community abundances associated with inner layers of bentonite samples over the 7 years. While microbial abundances did not change in the inner layers, the PLFA data suggest potential changes in microbial community composition and could also indicate prolonged microbial turnover rates. Overall, the results support microbial stability in compacted bentonite exposed to DGR-like conditions for at least 7 years.

**IMPORTANCE:**

Long-term assessments of changes in microbial activity in compacted low-biomass bentonite systems analogous to deep geological repositories (DGRs) are critical to test conditions for stable engineered bentonite barrier components. This study assesses long-term temporal changes in microbial communities of compacted bentonites exposed to natural groundwater. It offers 7-year data that indicate stability of bentonite-based materials intended for use in engineered barrier systems of a DGR for the safe, long-term disposal of used nuclear fuel, with wider implications for microbial persistence in a deep subsurface environment.

## INTRODUCTION

Deep geological repositories (DGRs) rely on a multi-barrier system for the safe, long-term disposal of used nuclear fuel for hundreds of millennia. This multi-barrier approach utilizes the engineered barrier system (EBS) where the used nuclear fuels are encased in metal containers that are embedded in highly compacted bentonite boxes to be emplaced at least 500 m from the ground surface (1–11). Microorganisms could compromise DGR integrity via metal container corrosion, transformation of bentonite buffer mineralogy, gas production, and/or sorption and subsequent mobilization or immobilization of radionuclides (12–15). Microbially influenced corrosion of the metal container is an area of interest as it could impact the long-term integrity of the container over thousands to millions of years (16). Short-term studies suggest limited corrosion of candidate metal materials under DGR-like conditions (14, 17, 18), but data are limited. Therefore, assessing temporal changes in microbial abundance, diversity, and activity within bentonite materials is critical for building confidence in long-term performance of bentonite-based engineered barriers in a DGR.

The Materials Corrosion Test (MaCoTe), at the Grimsel Test Site in Switzerland, is an international project designed to assess the effectiveness of materials intended for use in EBS in a DGR for the safe and long-term management of high-level nuclear waste (14). This *in situ* long-term experiment provides an opportunity to better understand microbial community composition and dynamics in low-biomass bentonite systems while providing data critical for a successful DGR design. The test site is located 450 m beneath the surface of Juchlistock mountain, at an altitude of 1,730 m above sea level in the granitic Aar massif formations. In the experiment, metal container candidate materials (i.e., carbon steel, stainless steel, and copper coupons) were embedded in eight modules of two dry densities (1.25 or 1.50 g cm^−3^) of Wyoming MX-80 bentonite (American Colloid Company, USA) and exposed to natural groundwater in a 9 m borehole (14). The groundwater chemistry is previously reported, where groundwater temperature ranged between 14°C and 16°C, pH between 8.2 and 8.4, electrical conductivity between 0.1 (in 2014) and 4.1 mS cm^−1^ (in 2022), and redox potential (Eh) between –60 and –155 mV (12).

Relatively short-term prior experiments suggest limited microbial abundance and activity in compacted bentonite likely due to unfavorable conditions such as limited hydraulic conductivity and low water activity due to compaction, and sorptive effects of bentonite (11–13, 19). Leveraging MaCoTe samples, we assessed microbial abundance and diversity profiles in compacted bentonite samples over 7 years of module emplacement. Modules were retrieved at years 1, 5, and 7 after emplacements, and the bentonite samples were separated into inner and outer layers (see Materials and Methods for details). The results for PLFA analyses, complemented by 16S rRNA gene quantification and sequencing, and cultivation approaches, for samples collected after years 1 and 5 of emplacement, indicating no significant changes in microbial communities, were previously published (12, 19). Herein, we present data for samples collected 7 years after emplacement in relation to the starting material (year 0) and years 1 and 5 results. We also assessed changes in natural organic matter (NOM) composition in the bentonite samples to evaluate potential microbial use of this pool and relate any shifts in microbial populations to changes in NOM quantity and quality. The insights obtained from this study will also help evaluate the effectiveness of our multidisciplinary approach in assessing microbial systems in low-biomass and low-nutrient environments. It will not only inform the long-term performance of bentonite-based engineered barriers in a DGR but also enhance our understanding of microbial habitability under extreme subsurface environmental conditions.

## RESULTS

### PLFA abundance and diversity in year 7 bentonite samples

**Fig 1:**
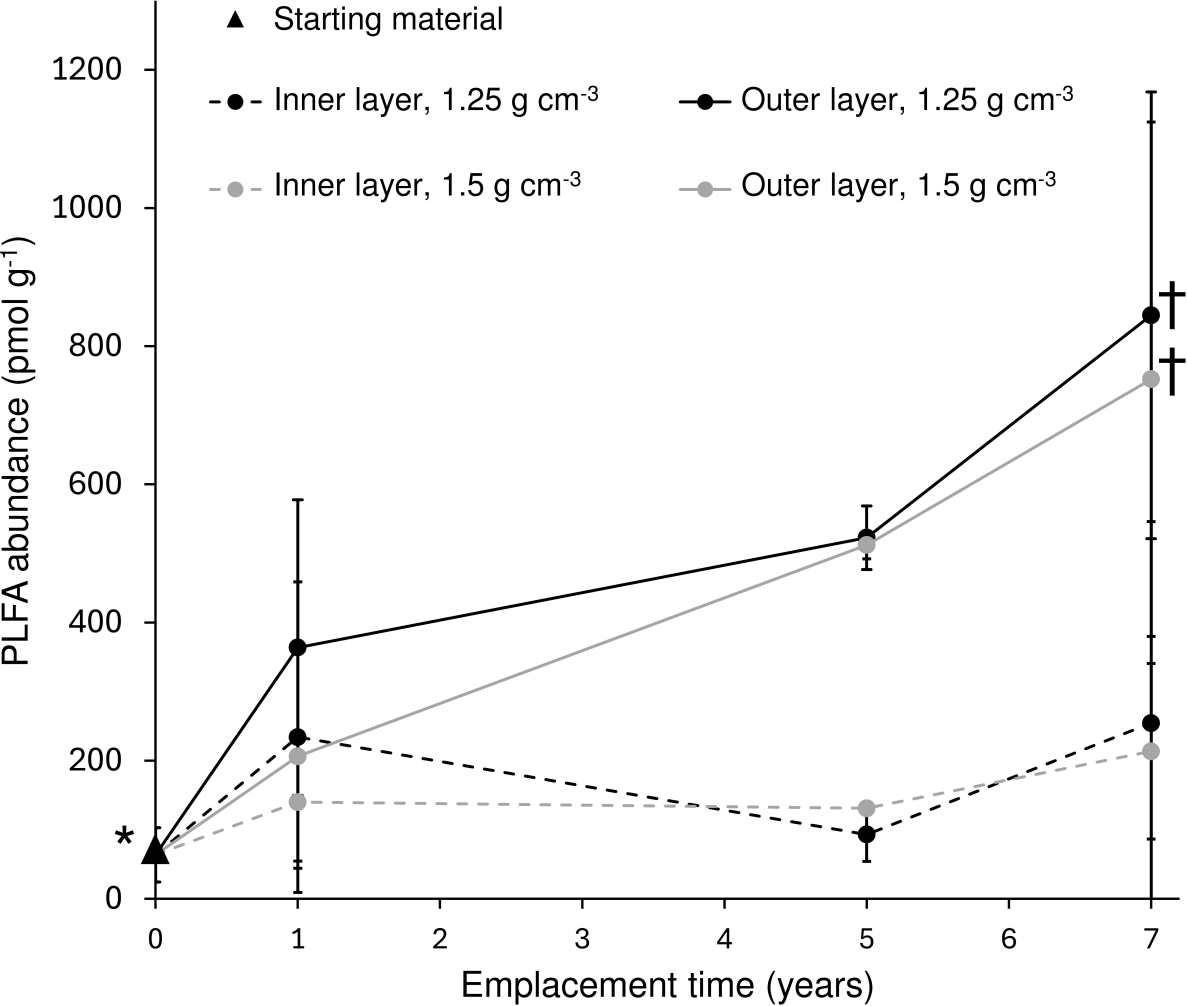
PLFA abundances (pmol g^−1^) in the inner and outer layer bentonites from low (1.25 g cm^−3^) and high (1.50 g cm^−3^) dry density modules emplaced in borehole 13.001 for 0, 1, 5, and 7 years. Error bars represent SD around mean for samples belonging to a particular layer, density, and year. The symbols († and *) indicate statistically higher pmol g^−1^ PLFA in the year 7 outer layer samples (for both densities) compared with the starting material where starting material represents average (±SD) of samples analyzed along with both year 5 and year 7 modules (two replicates each; total four replicates). Data for years 1 and 5 were previously reported (12, 19).

Total PLFA concentrations for year 7 inner layer samples were 250 ± 290 pmol g^−1^ and 210 ± 130 pmol g^−1^ for the 1.25 g cm^−3^ and 1.50 g cm^−3^ dry density modules, respectively (Fig. 1; Table S1). The PLFA concentrations in the year 7 outer layers were 850 ± 320 pmol g^−1^ (1.25 g cm^−3^ density) and 750 ± 370 pmol g^−1^ (1.50 g cm^−3^ density). No significant difference was observed between 1.25 and 1.50 g cm^−3^ density samples and between inner and outer layers. The PLFA abundances in the inner layer samples at both densities were statistically similar to those in the starting material (64 ± 39 pmol g^−1^), indicating no change in PLFA abundances in the inner layer over a 7-year time span. Only the PLFA abundances in the year 7 outer layer samples were statistically higher than those in the starting material and only when the starting material analyzed along with both year 5 and year 7 was considered in aggregate (Fig. 1).

Up to 44 distinct PLFAs, ranging from C10 to C28 in carbon chain length, were detected in the year 7 bentonite samples. The PLFA profiles in all samples were dominated by general bacterial markers (i.e., short-chain saturates, mostly 14:0, 15:0, 16:0, and 18:0, and short-chain monoenoics, mostly 18:1; Fig. 2). Together, short-chain saturates C16:0 and C18:0 accounted for 31% to 42% of the total PLFAs detected, followed by short-chain monoenoic C18:1ω9 at 9% to 14%. Other PLFAs, including short-chain saturates C14:0 and C15:0, short-chain terminally branched iso and antiso C15:0 and iso C16:0, short-chain monoenoic C16:1ω9, and short chain polyenoic C18:2ω9,12, represented 5% or less of the total PLFAs. The predominance of saturated PLFAs is indicative of low-biomass environments (20). Many samples also contained up to 22% of PLFAs with 20+ carbon chain length, which are often produced by eukaryotes such as fungi (20, 21), though the 18S rRNA gene sequencing analysis detected no abundant fungal amplicon sequence variants (ASVs) in bentonite samples (data not shown). The number of total detected PLFAs was unaffected by the bentonite density and whether the samples were from the inner or outer layer (Table S1). Pairwise Spearman’s rank correlation analysis of PLFA profiles in year 7 samples generally showed moderate to strong correlations with no discernable differences based on sample layer or density (Fig. S1).

**Fig 2:**
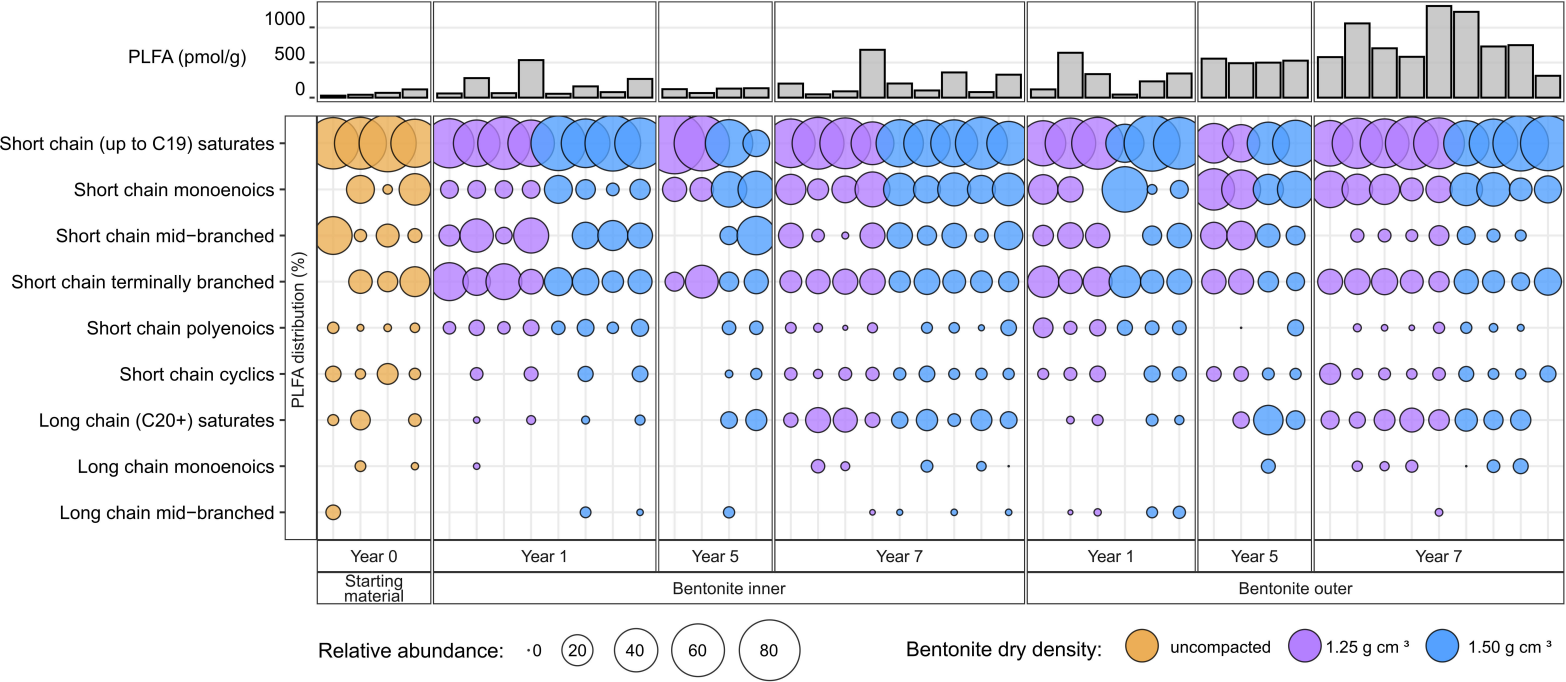
Relative abundance (mol%) of individual PLFAs in each bentonite sample and total PLFA (pmol g^−1^) shown at the top. Data for years 1 and 5 were previously reported (12, 19).

### Microbial abundances and diversity in year 7 bentonite samples

**Fig 3:**
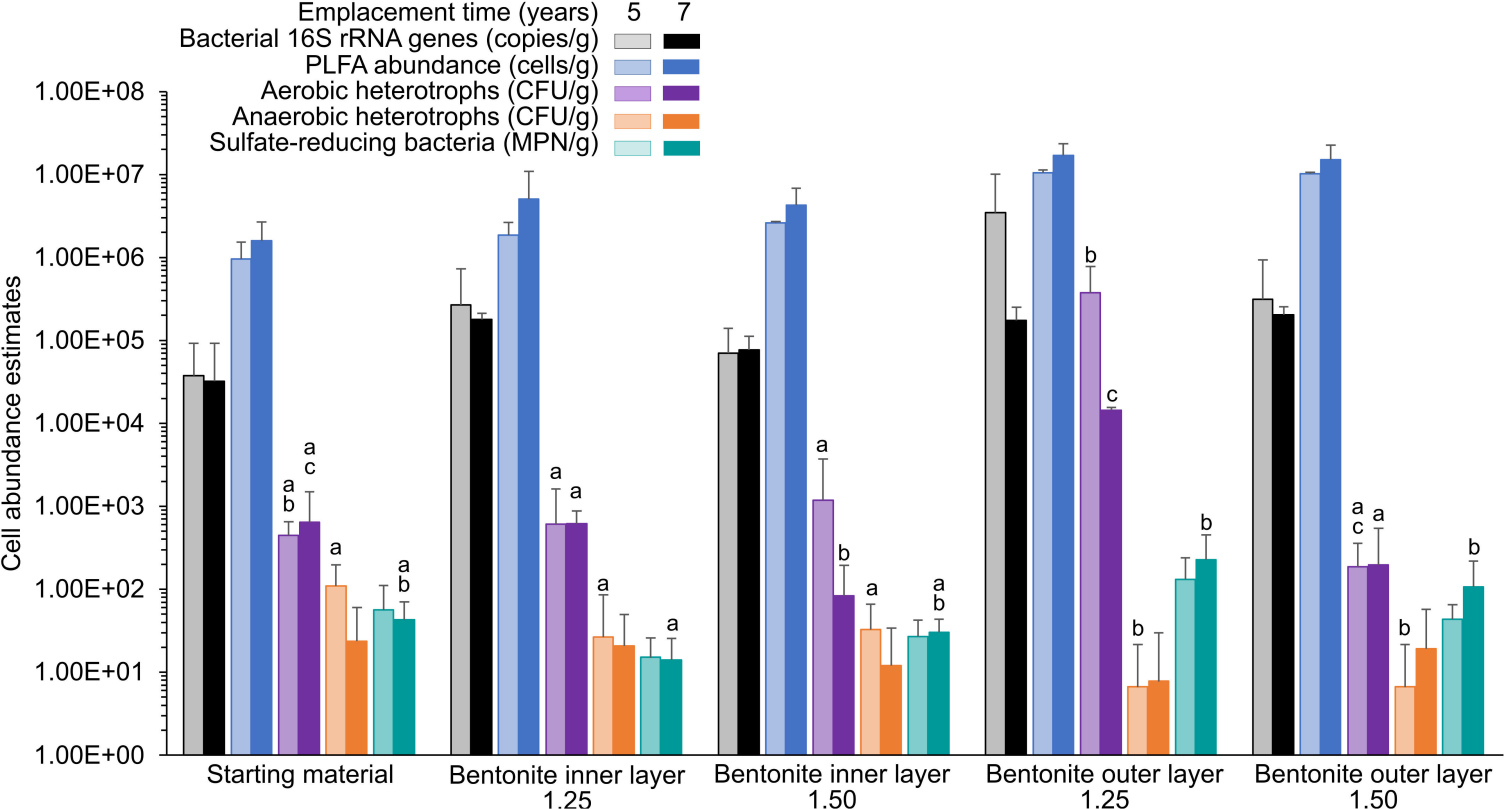
Cultivation, bacterial 16S rRNA gene qPCR, and PLFA analysis-based microbial abundance estimates in outer and inner layers of borehole module bentonite samples from years 5 and 7 in comparison to starting material. Year 5 data were previously reported (12). Cultivation data for year 1 samples were generated by another lab (22), and therefore, we did not include year 1 data herein. Starting material analyzed along with year 5 and year 7 samples (two replicates each) is presented separately to show no significant year to year material or analysis methodology-related differences in the data. Error bars represent SDs based on two to five replicates (PLFA) or five replicates (16S rRNA and cultivation data). Statistics is based on Dunn’s test with Benjamini-Hochberg *P* value adjustment (*P* < 0.05; after a significant Kruskal-Wallis test). Different letters (a, b, and c) above bars indicate significant differences within the abundances obtained from a specific analysis method and year, including starting material analyzed along with bentonite samples from that specific year (i.e., comparisons are across same-colored bars). No differences in PLFA-based microbial abundances are observed in any sample likely due to the use of a lower number of replicates of the starting material.

Microbial abundances based on the PLFA analysis were 5 × 10^6^ (1.25 g cm^−3^) and 4 × 10^6^ (1.50 g cm^−3^) for the inner layers of the compacted bentonite samples and were statistically similar to the starting material (Fig. 3; Table S9). The PLFA-based microbial abundances in the outer layers were 2 × 10^7^ (for each density), values 4 to 5 times yet statistically similar to values in the inner layers. The outer layer values were also statistically similar to the starting material when assessing changes within a given year’s data set, i.e., when we compared PLFA-based abundances for year 7 bentonite samples and the starting material (two replicates) analyzed along with these samples (Fig. 3). The abundances in the outer layers were, however, statistically higher than those in the starting material when all the replicates of the starting material (i.e., two replicates analyzed along with year 5 and two replicates analyzed along with year 7; total four replicates) were included (Fig. 1; Table S9). The starting material was analyzed along with year 5 and year 7 bentonite samples to assess year to year material and analysis methodology-related variations, which were minimal as shown in Fig. 3. The significant PLFA result is likely due to greater statistical resolving power obtained by combining all replicates of the starting material. The increase in PLFA-based abundances in the year 7 outer layer compared with the starting material is likely also small as all four replicates of the starting material were needed to reach significance. Culture-based abundance estimates of anaerobic heterotrophs, aerobic heterotrophs, and SRB ranged between ~10 and 50 CFU g^−1^, 10^2^ and 10^5^ CFU g^−1^, and 10 and 500 most probable number (MPN) g^−1^, respectively. Although the abundances of anaerobic heterotrophs and sulfate reducers were statistically similar for the inner and outer layers of both bentonite densities, the aerobic heterotroph abundances for 1.25 g cm^−3^ outer layer samples were orders of magnitude greater than those in the 1.50 g cm^−3^ outer layer and both inner layer samples. The bacterial 16S rRNA gene copies of inner and outer layer samples of both densities were approximately 10^5^ copies g^−1^ and were not statistically different than the starting material (Fig. 3). The estimated abundances varied based on the detection method, and the PLFA-based values were greater than 16S rRNA gene quantitative PCR (qPCR)-based values.

**Fig 4:**
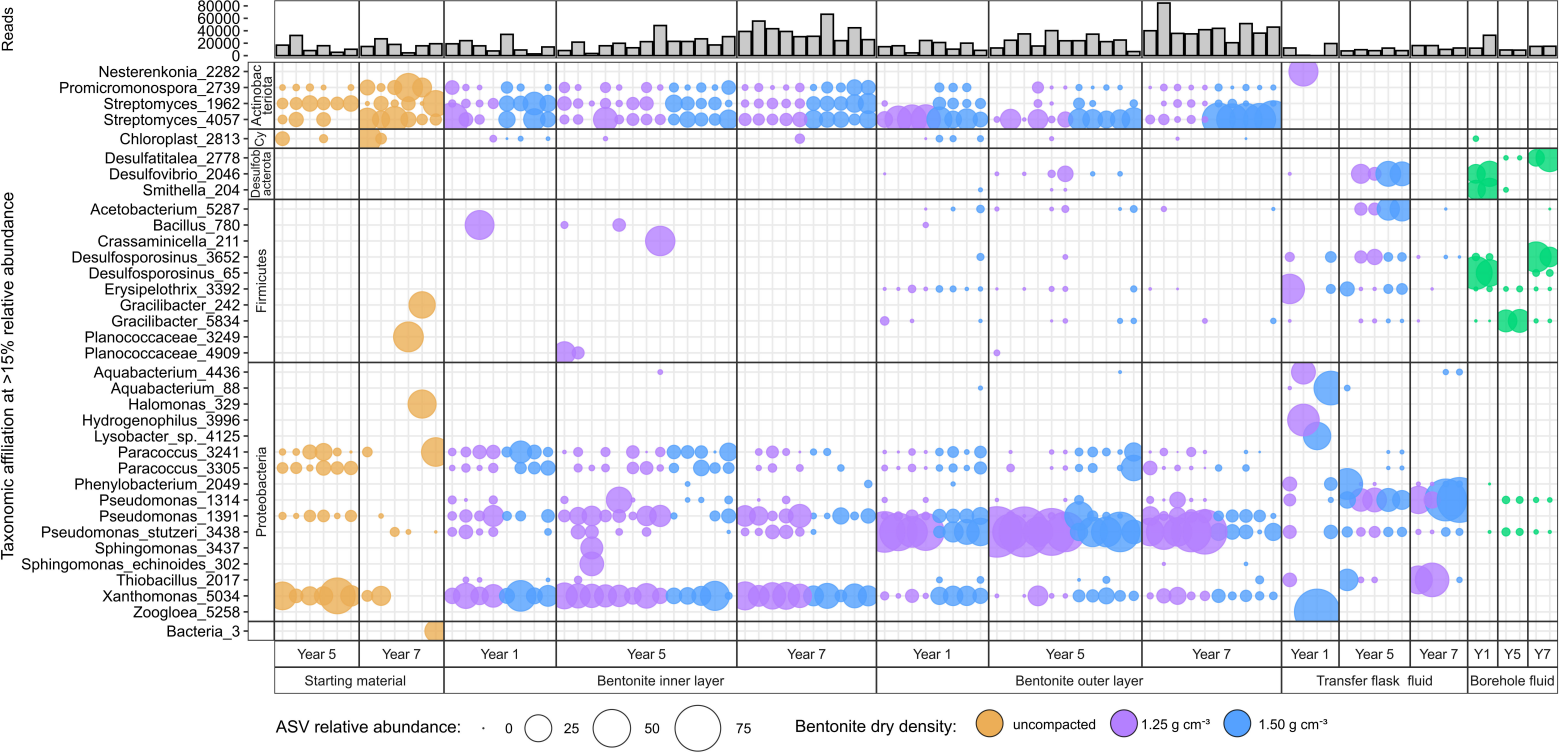
Bubble plot showing 16S rRNA gene profiles of the starting material and year 1 to 7 borehole module bentonite samples from outer and inner layers. 16S rRNA gene profiles of borehole and transfer flask fluids from years 1, 5, and 7 are also shown. Starting material was analyzed along with year 5 and year 7 samples. Data for years 1 and 5 were previously reported (12, 19). Only ASVs at or above 15% relative abundance are shown. ASVs and their phylum association are shown on the left with “Cy” representing Cyanobacteria.

**Fig 5:**
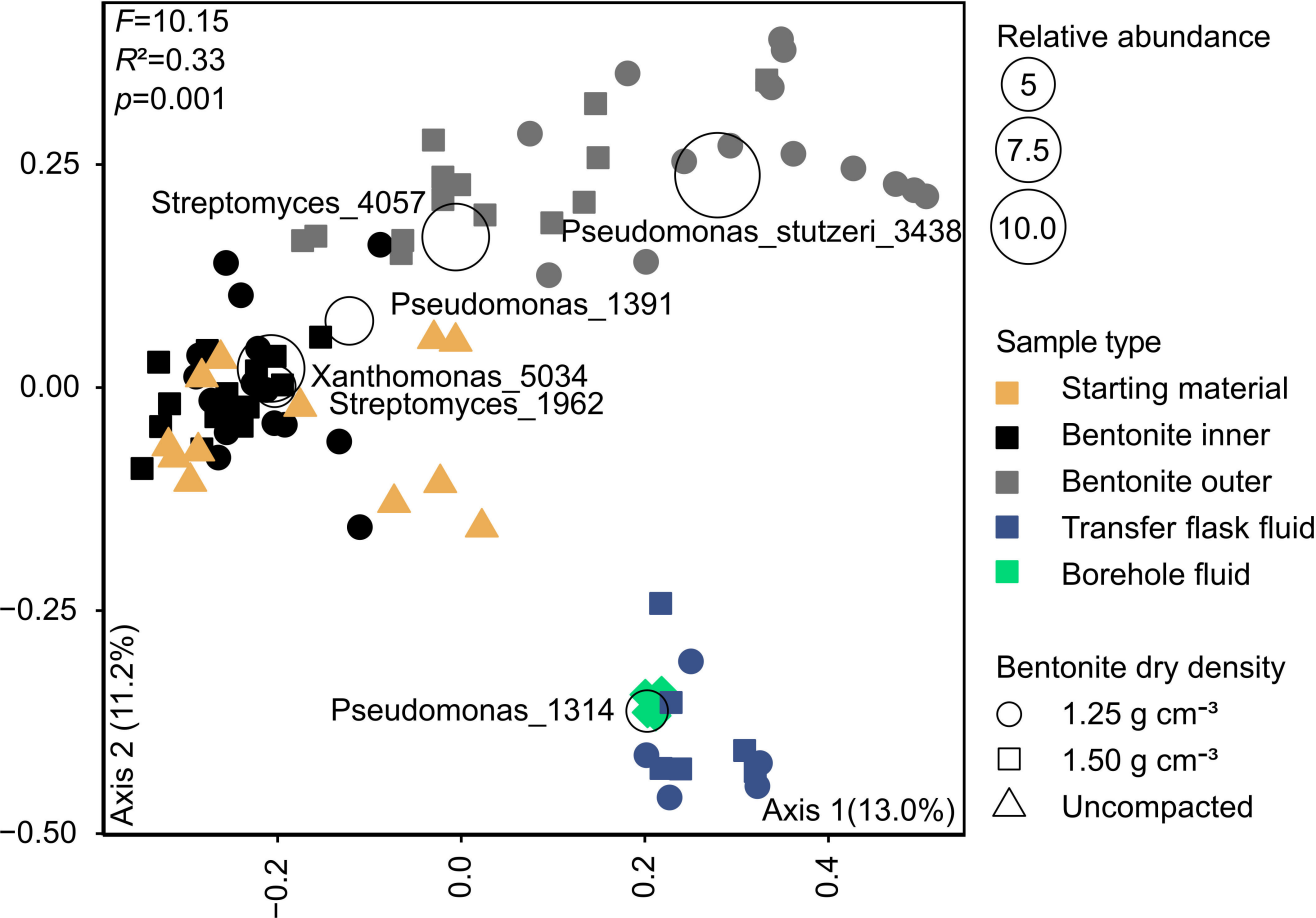
Grouping of years 1 to 7 borehole module samples, borehole fluid, transfer flask fluid, and starting material in a principal coordinate analysis ordination based on Bray-Curtis metric. Data for years 1 and 5 were previously reported (12, 19). ASVs at or above 2% relative abundance are shown. Samples were rarefied to 3,000 reads. The *adonis* function in QIIME2 was used to determine statistical significance between bacterial communities across sample groups.

The 16S rRNA gene profiles of the inner and outer bentonite layers indicated differences in bacterial composition (Fig. 4 and 5). *Xanthomonas* and *Streptomyces* were the most abundant ASVs in the inner layers for both densities. *Pseudomonas stutzeri* was detected in many inner layer low-density samples but only in three of the high-density samples. *Pseudomonas stutzeri*, *Streptomyces*, and *Xanthomonas* ASVs were most abundant in the outer layer samples, whereas the low-density samples were dominated by *Pseudomonas stutzeri* and the high-density samples by Streptomyces ASVs (Fig. 4). The detected PLFA profiles (Fig. 2) could be generally attributed to all the identified ASVs and do not provide sufficient information to support the observed ASV differences between different samples. Although many samples contained PLFA markers (C18:2ω9,12 and PLFAs with 20+ carbons) often associated with eukaryotic organisms like fungi and plants (20, 21, 23), we did not target these microorganisms with molecular methods in this study.

### Microbial community profile comparisons for years 0, 1, 5, and 7 bentonite samples

Overall, the PLFA, cultivation, and 16S rRNA gene data suggest minimal changes in the inner layer samples for both bentonite densities over the 7 years of borehole module emplacement. However, the PLFA and aerobic heterotroph abundances and the 16S rRNA gene profiles of outer layer samples were different from the starting material after 7 years (Fig. 1 and 3 to 5). The total PLFA abundances in the outer layer samples at both densities increased and became statistically greater than those in the starting materials at the 7-year time point when all four replicates of the starting material were included (Fig. 1). Principal coordinate analysis (PCoA) showed that the starting material and inner layer of both densities grouped together (Fig. 5), though both groups were significantly different based on permutational multivariate analysis of variance (PERMANOVA) (*P* = 0.002; Table S10). Phylogenetic diversity of the inner bentonite layer samples was statistically higher compared with the starting material for year 7 samples as well when year 1 to year 7 samples were analyzed combined (Fig. S7). The 16S rRNA gene profiles from the inner layer samples remained similar temporally and to the starting material (Fig. 4; Fig. S4 and S5). The dominant ASVs in year 1 outer layer samples were different compared with those in the starting material and underwent further changes over 7 years, indicating a more dynamic system. Phylogenetic diversity of the year 7 outer bentonite layer was statistically higher compared with the starting material (Fig. S7). At the 7-year interval, the relative abundance of *Streptomyces* taxa decreased in the low-density samples whereas they increased in the high-density outer layer samples (Fig. 4 and 5). Abundances of anaerobic heterotrophs in the year 5 and 7 samples were statistically similar to or lower than those in year 0 samples. For SRB abundance estimates, no statistical difference between the starting material and inner and outer layer year 5 and 7 bentonite samples was observed. The cultivation results were also similar to year 1 data reported in reference 22. Only very few putative SRB ASVs were identified in the starting material and inner bentonite samples, with relative abundances below 1.5% except for one starting material replicate, which showed 11.2% relative abundance of an ASV associated with *Desulfosporosinus* (Fig. S7). Outer bentonite year 1 and 7 samples appear similar to inner bentonite samples with only few putative SRB ASVs present at low abundances and often only present in one or few replicates. In contrast, relative abundance of putative SRB was higher for year 5 outer layer bentonite, and some were present in several replicates such as *Desulfatitalea* ASV #1270, *Desulfocapsaceae* ASV #315, *Desulfovibrio* ASV#2046, and *Desulfosporosinus* ASV#1520. Majority of putative SRB ASVs abundant in year 5 outer layer were also detected in the transfer flask fluid of the same year, but many were absent in the year 5 borehole fluid. Aerobic heterotroph abundances were higher in the low-density outer layer year 5 and 7 samples, though not statistically significant for the later year. The Spearman rank correlation and Kruskal Wallis test of individual PLFA profiles (Fig. S1; Tables S2 and Table S3) suggest some differences in PLFA profiles between years 0, 1, 5, and 7 samples (even for the inner layer samples), indicating potential shifts in microbial communities over time. However, one-way analysis of variance (ANOVA) indicated no differences among PLFA profiles. Furthermore, the culture and 16S rRNA gene data do not indicate significant microbial community shifts for the inner layer samples. Therefore, any changes occurring in the PLFA profiles are likely very small, close to our current ability to resolve them.

### NOM analysis

Overall, there were no marked changes in organic carbon or the NOM chemistry (SI Section II). The inorganic carbon content was statistically similar in both density samples over the 7 years, but the organic carbon content in the low-density samples was slightly lower in the year 7 samples (0.23% ± 0.02% in the starting materials versus 0.18% ± 0.01% in the year 7 samples; Table S5). This small difference is within the range of heterogeneity reported for MX-80 (24). As such, the observed difference does not exceed the threshold for variability in MX-80 bentonites. Solid-state ^13^C nuclear magnetic resonance (NMR) results indicate that the NOM makeup of bentonite samples was similar to other Wyoming-type bentonites and did not change for either bentonite density over the 7 years (SI Section II; Table S4). The majority (~50%) of NOM in the analyzed samples consisted of relatively persistent alkyl carbon components that are not preferred microbial substrates (Table S4). The remainder of NOM was predominantly aromatic (~25%), followed by O-alkyl carbon (~15%) and carboxylic and carbonyl carbon (~8%; Table S4).

## DISCUSSION

In this study, we assessed long-term trends of temporal shifts in microbial abundance and diversity of bentonite modules in the subsurface environment of the MaCoTe project. The long-term nature of this experiment and the use of several complementary microbial analysis techniques provide a unique opportunity to improve our understanding of the behavior of bentonite-associated microbial communities in low-biomass, low-nutrient subsurface systems analogous to a DGR. Overall, the culture-dependent and independent analyses conducted herein indicate minimal changes in microbial abundances and diversity in the inner layer bentonite samples, which are most relevant to the DGR design because of proximity to the nuclear fuel cannisters, over the 7 years of module emplacement (Fig. 1 to 5; Fig. S1, S4 and S5; Table S1). The results support our previous work and suggest that the high-density bentonite sufficiently suppresses overall microbial growth and activity (12, 19) for at least 7 years and likely much longer. A similar experiment (25) also suggests microbial stabilization in compacted bentonite for at least several years after module deployment. Furthermore, the microbial abundances determined in this study are within the range of values published for similar approaches involving shorter term experiments and similar type of bentonites and dry densities (11, 25).

Microbial abundances detected in the inner layer samples did not change over the 7 years (Fig. 1 and 3; Table S1). In contrast, the microbial abundances of outer layer samples increased, likely due to exposure of the outer surface to the surrounding natural groundwater, allowing microorganisms to use nutrients from the groundwater (water composition available in reference [26]) while inhabiting an interface with potentially lower dry density conditions. It appears that the dominant groundwater microorganisms did not colonize the outer layers but likely microorganisms present in the bentonite proliferated at the interface due to more favorable conditions. This is supported by the moisture content and water activity differences between the two layers where the values in the outer layers were greater than those in the inner layers (Table S6). The higher the moisture content and water activity, the more microbial growth is expected (27), which could explain the increase in microbial abundance in the outer layers. ASVs associated with *Streptomyces*, *Xanthomonas*, *Paracoccus*, and *Nocardiopsis* were absent from the borehole fluid, and their origin from the starting material is likely. Even though *Pseudomonas stutzeri* ASV #3438 was present in the borehole fluid and the starting material, its origin from the latter appears likely as *Pseudomonas stutzeri* was cultivated from the starting material previously (12). Literature also supports minimal colonization of compacted bentonite by groundwater microbes (25, 28) and enhanced proliferation of microbes at outer surfaces due to relatively favorable conditions (11, 13, 29). The actual dry densities for the outer and inner bentonites were not measured after emplacement, but the detected moisture content and water activity differences between both compartments could be indicative of actual density difference. Because the outer layer samples comprised only a ~0.5 cm thin layer of the bentonite modules, potential heterogeneity and variability should be considered while interpreting these results. Due to the consistency of the year 1, 5, and 7 outer bentonite 16S rRNA gene profiles and the presence of those ASVs at low abundance inside the bentonite core, it is unlikely that those profiles originate from contamination during dismantling, handling, and subsampling.

The 7-year inner layer samples contained ASVs affiliated with *Xanthomonas*, *Streptomyces*, *Pseudomonas*, and *Paracoccus* (Fig. 4). These ASVs were also abundant in the starting materials and have been previously reported for MX-80 bentonite (28, 30, 31). A total of 12 starting material replicates were analyzed, and some variations for the relative abundance of dominant ASVs were observed. These variations could be attributed to extraction efficiencies, inherent low biomass, and heterogeneity of the material. The starting material is stored under oxic conditions, at room temperature, which could contribute to changes over time. The ASVs in the inner layer samples remained generally similar over the 7 years (Fig. 4 and 5). The outer layer samples were dominated by ASVs affiliated with *Pseudomonas stutzeri* and *Streptomyces* starting year 1 and then generally remained similar up to 7 years for the low-density module. These results suggest that microbial communities in the inner versus outer layers shifted early after module deployment and then stabilized. This early change in the community composition in the outer layers could be attributed to greater exposure of the module surface to ambient natural water and its nutrients and the potentially lower bentonite density at the interface, which enabled some microorganisms in the starting materials to proliferate in the outer layers. Cultivation data showed viable aerobic heterotrophs in bentonite samples exposed to anaerobic conditions for up to 7 years, but facultative anaerobes such as *Pseudomonas* will also be captured in those cultivation conditions. Aerobic microorganisms such as *Xanthomonas* were likely dormant and inactive during emplacement time, whereas *Pseudomonas stutzeri* likely proliferated in the outer layer bentonite during early stages of module emplacement as no increase in relative abundance was observed after year 1. Persistence of aerobic microorganisms in anoxic compacted bentonite was observed previously but not attributed to facultative anaerobes as the number of cultivated aerobes exceeded anaerobes (25). Facultative anaerobes may also be underrepresented in anoxic heterotroph incubations due to missing electron donors or acceptors (e.g., nitrate for denitrification) as *Pseudomonas stutzeri* was previously not enriched on anoxic R2A agar incubations despite being dominant in oxic incubations (12). The shift of relative abundances of *Streptomyces* ASV 4057 and *Pseudomonas* ASV 3438 in high-density outer layer bentonite samples could be attributed to active growth of *Streptomyces* or density-related reduction of *Pseudomonas* taxa. However, the representative sequence of ASV 4,057 matched 100% to aerobe, spore-forming Streptomyces strains, making it unlikely to grow actively in an anoxic environment. Considering no increase in cultivable microorganisms was detected over time, it appears more likely that *Pseudomonas* cells die, their genomic DNA degrades, resulting in lower relative abundances in 16S rRNA gene profiles while spore-forming Streptomyces are more resilient to degradation.

Minimal changes in microbial abundances and diversity in the inner layers over the 7 years could be due to (i) very slow biomass turnover rates in a nutrient deficit environment which can send microorganisms into prolonged maintenance/static mode (32, 33) and/or (ii) long-term preservation of dead microorganisms and their contents (e.g., PLFAs) in compact, sorptive bentonite materials because of limited biotic (due to lack of active microorganisms) and abiotic (due to poor water activity and movement and organism sorption onto clay particles) degradation. While both scenarios might prolong the presence of intact polar lipids (IPLs; including PLFAs) in such environments, the latter option means that IPLs that are often thought to indicate viable microorganisms in biologically active surface systems (34–37) may not necessarily represent living cells in biologically less active and compact environments, such as deep subsurface. Potential changes in PLFA profiles of the inner layers over time (Fig. 2; Fig. S1; Table S3) suggest that prolonged biomass turnover rates might be stabilizing microbial abundances herein. Although natural heterogeneity and variability cannot be ruled out, these changes could indicate the presence of active microorganisms metabolizing and growing at extremely slow rates because of a high-density, nutrient-deficient environment. Cultivation data reveal cultivable microorganisms in the samples. Previous studies also suggest the presence of slowly metabolizing or potentially dormant microorganisms under similar conditions (1, 11). Persistent microorganisms have been detected in various deep subsurface formations that can be millions to billions of years old (38–40). Microbial turnover rates in these similar (i.e., compact, low biomass, and nutrient poor) subsurface environments have been estimated to range from centuries to millions of years (41–43), where compaction restricts microbial movement and further limits the availability of nutrients and electron acceptors in an already-nutrient-poor environment (41, 44). These dormant or slowly metabolizing microorganisms are relevant to long-term stability of the DGR because significant disturbances to the EBS (e.g., significantly reduced compaction) could potentially create conditions favorable for their proliferation. As such, DGR designs must ensure long-term maintenance of the high-density, nutrient poor, unfavorable conditions.

The PLFA data indicate statistically similar microbial abundances in inner and outer bentonites for both 1.25 g cm^−3^ and 1.50 g cm^−3^ dry densities associated with year 7 modules. Culture-based data indicate some differences (Fig. 3), showing greater aerobic heterotroph abundances for 1.25 g cm^−3^ outer layer samples at year 7, which was similar to results observed for the 1.25 g cm^−3^ outer layer samples at year 5 (Fig. 3) and year 1 (22). Culture-based enumeration yielded the lowest abundance counts as only specific metabolic groups such as sulfate-reducing microorganisms were targeted and not all microorganisms present in a sample are cultivable under conditions provided in the laboratory, usually leading to an underestimation when using this method. In contrast, the primer pair used for qPCR is designed to target the majority of all bacterial groups; however, the method cannot differentiate between live and dead cells and results could be inflated due to multiple gene copies in some microorganisms. Cultivation-based, DNA-based, and PLFA-based methods do not always yield comparable abundance estimates due to method limitations but are complementary and together offer a more detailed picture of microorganisms present and/or viable.

No increase of cultivable SRB was observed within the bentonite core for both, the low- and high-density modules, after 5 and 7 years of emplacement, which suggests the survival of those SRBs as spores or inactive cells but no proliferation at either dry density. No change of the 16S rRNA gene profiles for inner bentonite samples over 7 years further suggests no activity within the bentonite core, which would include non-cultivable microorganisms. Comparably more ASVs associated with putative SRBs were identified in year 5 low- and high-density outer bentonite, though the SRB MPN estimates were not different compared with the other tested samples. This could indicate the presence of non-cultivable SRBs or DNA from dead cells, but it is not clear why the year 5 outer layer samples differ from those of years 1 and 7. Dominant ASVs in the transport container fluid were often not dominant in borehole fluid or outer layer bentonite for all years, which could indicate microbial activity and changes during the transport and storage within the transport container. This would emphasize the importance of quick processing of borehole modules after retrieval and storage at borehole relevant temperatures. However, the 16S rRNA gene profiles for the outer layer bentonite were consistent for all years, which indicates them being less affected by transport container changes. This observation can also be applied to the borehole fluid. Even though temporal changes can be observed for the 16S rRNA gene profiles, they appear to have no effect on the outer bentonite.

The relative abundances of *Streptomyces* ASV 4057 and *Pseudomonas stutzeri* ASV 3438 changed between year 1 and year 7 showing a decrease of *Streptomyces* and increase of *Pseudomonas stutzeri* in the low-density outer layer samples (Fig. 4 and 5). Together, these results suggest influences of bentonite density differences on microbial abundance and diversity. Moreover, the moisture contents and water activities (Table S6) in the high-density samples (both layers) were lower than those in the low-density samples. This, along with the 16S rRNA gene and aerobic heterotroph culture data, suggests that the high density, which is analogous to the density of natural bentonitic subsurface environments, inhibits microbial growth, even though water activity was relatively high at 0.96 (Table S6). Most bacteria are expected to grow at water activities above 0.96, though prolonged exposure of *Desulfomicrobium baculatum* and *Desulfovibrio* sp. to 0.96 and 0.98 water activities ceased their cultivability (5). These results are consistent with lab-based studies indicating decreasing microbial abundances with increasing bentonite densities (1, 5, 11). Literature suggests dry densities around 1.3 to 1.5 g cm^−3^ limit activity and survivability of sulphate reducers (1), whereas dry densities ≥ 1.6 g cm^−3^ are ideal to limit activity of most indigenous microorganisms (11, 29, 45). Although bentonite dry density affected some microbial measures in the present study, the different approaches used herein (i.e., PLFA, 16S rRNA, and cultivation) show lack of significant growth in the inner layers of both 1.25 and 1.50 g cm^−3^ densities over the 7 years. The present study indicates that even 1.25 g cm^−3^ bentonite dry density limits microbial survival and activity. Although more research is warranted to confirm this, the stabilization of indigenous microorganisms at relatively low dry densities has an implication for long-term performance of EBS, as heterogeneity of bentonite density would likely not significantly affect the growth of microorganisms. Increase in microbial abundance and activity at the interfaces of rock and bentonite could be of concern, as microorganisms such as SRB could produce sulfide that may diffuse through the bentonite layers and corrode the copper-coated containers (46). Short-term laboratory or modeling-based assessments suggest strong sorption of sulfides to bentonite and limited diffusion to metal containers (17, 18, 47). In this study, only very few putative SRB ASVs were detected in the outer layers over the 7 years, and there was no increase in cultivable SRBs, which could inform future experimental and/or modeling efforts to assess implications of microbial growth at EBS surfaces.

Another way to assess potential microbial growth and turnover is via evidence of metabolism of organic carbon sources present within the materials. Although the NOM composition of the inner layer samples did not change over 7 years for either density, the organic carbon content in the low-density bentonite was lower. Even though the low-organic carbon content (~0.2%) and relatively limited number of samples analyzed imply that heterogeneity may be a factor, the decrease in the organic carbon is also consistent with very slow microbial activity over the 7 years. However, the microbial community comprises a very small component of the NOM. Assuming the average bacterial cell mass of 10^−12^ g, microbial abundances on the order of 10^7^ g^−1^ based on the PLFA data (Fig. 3), and the average organic matter content of 0.2% (Table S5) in the modules, the microbial biomass comprises <0.5% of the NOM detected. Furthermore, changes in microbial biomass over the 7 years in the low-density inner layer samples represent only 0.6% of the change in the organic carbon content in those samples, suggesting something other than microbial turnover might be largely contributing to the change in organic carbon content. Heterogeneity coupled with low-organic carbon content in all the samples is a likely explanation for the organic carbon content difference between the year 0 and the low-density inner layer year 7 samples as the differences observed do not exceed the threshold previously reported for organic carbon variability in different batches of MX-80. The nutrient content data (Table S7) for the starting material and composite inner layer year 7 samples indicate that the bentonite starting material is deficient in available phosphorus (sodium bicarbonate extractable P was ~9 mg kg^−1^), which could limit microbial growth. Compaction, however, likely plays a greater role in limiting growth than phosphorus or organic carbon availability. The phosphorus content in the inner layers slightly decreased to 8.8 (high-density samples) and 7.4 (low-density samples) mg kg^−1^ over the 7 years. Taking the organic carbon content data (Table S5) and slight decrease in available phosphorus over the 7 years (Table S7) into account, it is possible that slowly metabolizing microorganisms utilized a part of the decreased NOM for cellular maintenance. Microbes in compacted and saturated bentonite can consume organic matter (48). However, we have limited data to confirm if this is happening in our study. Mineralogy of the inner layers of the two densities remained similar to the starting material (see SI Section V and Table S8), indicating structural stability and no illitization after 7 years, as previously reported for a 1-year study (15). It would be interesting to assess NOM, nutrient, and mineralogical makeup of the outer layer samples as microbial abundances in them increased over the 7 years. Due to limited sample size, we were not able to conduct such analyses on the outer layers. Additionally, future studies should also assess NOM and nutrient composition of the groundwater as it could provide useful information about substrate availability to microorganisms in the outer layers. Unfortunately, these assessments were not conducted for the groundwater samples retrieved along with the years 1, 5, and 7 modules.

### Conclusions

The long-term *in situ* MaCoTe experiment being conducted at the Grimsel Test site presents an opportunity to assess the long-term fate of IPLs and microorganisms in compacted, low-biomass, nutrient-poor environments analogous to the deep subsurface. Our data indicate that expected conditions in DGRs (i.e., compaction, low water activity and hydraulic conductivity, low biomass, and nutrient limitations) can restrict microbial activity, supporting the case for stability of bentonite-based engineered barrier components in a DGR. On a broader level, such deep subsurface conditions could also result in long-term stabilization and preservation of IPLs and microorganisms in subsurface environments where microbial turnover rates may be considerably prolonged. This has far-reaching implications, especially for IPLs and their derivatives as biosignatures in low-biomass systems, where their detection is often employed for the reliable indication of extant and active microbial communities. These results indicate that in environmental subsurface sediments analogous to the bentonite samples presented here, caution must be taken when interpreting the data as belonging to viable cells because of very slow microbial turnover rates and IPL and 16S rRNA gene analyses potentially detecting relic (preserved non-viable or dead) materials.

## MATERIALS AND METHODS

### Module set-up and sampling

The MaCoTe experiment, described previously (14, 19), involves long-term emplacement of eight modules containing test metal coupons in compacted bentonite (Wyoming MX-80) at two dry densities (1.25 or 1.50 g cm^−3^) in a 9 m long borehole. The different dry densities of the bentonite were prepared by mixing fine (MX6) and coarse (MX7) Wyoming MX-80 bentonites (American Colloid Company, USA). The density of 1.25 g cm^−3^ represents improperly placed or degraded bentonite buffer whereas 1.50 g cm^−3^ represents the target density of well-functioning buffer (14). The 1.50 g cm^−3^ dry density is also more representative of subsurface environments where quarried bentonites can have densities ranging from 1.5 to 1.8 g cm^−3^ (49).

The modules were placed into borehole 13.001 at Grimsel URL, Switzerland in 2014, and exposed to natural groundwater under predominantly anoxic conditions. Retrieval, transfer, and disassembly of the modules was described previously (14, 19). Briefly, two modules, one of each dry density, were retrieved from the boreholes under anoxic conditions at three different time points: years 1, 5, and 7 (394, 1,661, and 2,605 days of exposure, respectively). During disassembly, the bentonite cores were segmented into five sections (S1 [top] to S5 [bottom]). After sectioning, approximately 0.5 cm outer layer of each bentonite section was removed, resulting in inner and outer layer samples. Results for year 1 and year 5 modules were previously reported (12, 19). Herein, we analyzed bentonite samples from both density modules for year 7, along with the starting material (MX6 and MX7; their average referred to as year 0 or starting material) for comparison. Water activity and moisture content of the samples were analyzed as described in SI Section III. Samples for DNA, NOM, and PLFA analyses were frozen at −20°C until analysis. Samples for cultivation were stored for up to 6 weeks under anoxic conditions at 4°C.

### PLFA extraction and analysis

The bentonite samples were lyophilized for 24 hours and stored at −20°C until extraction. The extraction and analysis methods are detailed elsewhere (19). Briefly, samples (outer layer 1.6 to 6.9 g; inner layer 22.2–95.3 g) were crushed and lipids were extracted using a modified Bligh and Dyer protocol (50, 51) where an excess of dichloromethane-methanol-phosphate buffer (1:2:0.8 vol/vol) was used as extraction solvent. The lipid extracts were separated using silica gel chromatography followed by mild-alkaline methanolysis of the polar fraction containing PLFAs to convert PLFAs to more volatile and non-polar fatty acid methyl esters (FAMEs). The FAMEs were purified via secondary silica gel chromatography and concentrated to 100 µL in dichloromethane under nitrogen. Analysis was carried out on an Agilent 7890B gas chromatograph (GC) equipped with a DB5-MS column (30 m × 0.25 mm × 0.25 µm film thickness; Agilent Technologies) and coupled with an Agilent 5977B High Efficiency Source Mass Spectrometer (MS). One microliter of the FAME sample was injected into the GC via a splitless injector held at 300°C. Helium was used as the carrier gas with a flow rate of 1.4 mL min^−1^. The oven temperature program was as follows: initial hold at 50°C for 1 min, 20°C min^−1^ ramp to 120°C, 4°C min^−1^ ramp to 160°C, 8°C min^−1^ ramp to 300°C, and then hold at 300°C for 5 min. Total run time per sample was 35 min. The system was then equilibrated at 50°C for 1 min before subsequent sample injection. Ions were generated by a 71 eV electron beam and spectra recorded at two scans per second with an m/z scanning range of 50–450 atomic mass units (amu). The ion source and MS Quad were kept at 300°C and 150°C, respectively. Individual FAME peaks were identified using retention times, molecular weights, and spectra comparisons against the National Institute of Standards and Technology MS database and bacterial reference standards (Bacterial Acid Methyl Ester mix alone or along with Supelco 37 Component FAME Mix; Sigma-Aldrich). Contamination was assessed by running procedural blanks with each extraction and analysis procedure. Peak quantifications were performed using 5-point calibration curves made from select (C12-C23) FAME standards run in triplicate. Based on calibration curves, the practical quantitation limit (PQL) per PLFA ranged from 0.08 to 0.5 pmol_PLFA_ g^−1^. Detected PLFAs that were below the PQL were assigned a value of half the PQL. The *R*^2^ values for all curves were greater than 0.98. The PLFA concentrations are reported in pmol_PLFA_ g^−1^ (dry weight) sample. Cellular abundances (cells g^−1^) were estimated by applying a conversion factor of 2 × 10^4^ cells pmol_PLFA_^−1^ to the concentrations (52).

### Cultivation

Two grams (wet weight) of bentonite was suspended in 18 mL phosphate-buffered saline (PBS) and vortexed at low speed for 30 min at room temperature. Each suspension was subjected to 10-fold serial dilutions in PBS. Culturable aerobic and anaerobic heterotrophic bacteria were enumerated, in triplicate, on Reasoner’s 2A agar plates (R2A, M1687; HiMedia Laboratories). The plates were incubated at 30°C under oxic (7 days) or anoxic (28 days) conditions. For culturable SRB, suspensions were incubated for 28 days at 30°C under anoxic conditions and enumerated on lactate and sulfate containing SRB medium (M803; HiMedia Laboratories) by applying a five-tube MPN method. The average of five biological replicates was used for each enumeration of cultivable microorganisms. Cultivation of year 7 samples was performed in an anoxic chamber (Coy Laboratory Products). PBS was flushed with nitrogen gas for 30 min. Starting material was cultivated along year 5 and year 7 samples.

### DNA extraction

Total genomic DNA was extracted from 2 g bentonite sample using the DNeasy PowerMax Soil Kit (Qiagen, CA, USA) as per manufacturer’s instructions with the following modifications. The PowerBead tubes were incubated at 65°C for 30 min after addition of lysis solution, followed by bead beating using the MM 400 Mixer Mill (Retsch) at 30 Hz for 10 min. DNA was eluted in 2 mL elution buffer. DNA concentrations were measured on a Qubit 4.0 fluorometer (Life Technologies, CA, USA) using a Qubit dsDNA High Sensitivity Assay Kit (Invitrogen, CA, USA). Kit controls (i.e., no sample added) were included with each extraction batch. All bentonite DNA extracts were below the detection limit of the Qubit fluorometer.

### Quantitative 16S rRNA gene PCR

The abundance of 16S rRNA gene copies in the extracted DNA was estimated using technical duplicates on a CFX Opus Real-Time PCR System (Bio-Rad, California, USA) where 341F (CCTACGGGAGGCAGCAG) and 518R (ATTACCGCGGCTGCTGG) were used as primers (53). The reaction mixture (15 µL) comprised 1× SsoAdvanced Universal SYBR green Supermix (Bio-Rad, California, USA), 0.3 µM (each) forward and reverse primers, 7.5 µg bovine serum albumin (BSA, UV treated), and 2 µl DNA extract. The PCR cycle consisted of 98°C for 3 min initial denaturation, 40 cycles of 98°C for 15 s, and 55°C for 30 s. The standard curve was prepared from a 10-fold serial dilution of the 16S rRNA gene of *Thermus thermophilus* as described previously (54). The coefficient of determination (*R*^2^) was 0.99, and amplification efficiency was 108.4%. The qPCR estimates are based on an average of five biological replicates. The gene copy numbers were determined per gram of bentonite samples (dry weight).

### Amplicon sequencing

The V4–V5 region of the 16S rRNA gene was amplified using primers 515F-Y (55) and 926R (56). The 25 µL PCR mix contained 1× ThermoPol Buffer, 0.2 µM of each primer, 200 µM dNTPs, 15 µg BSA, 0.625 units Hot Start *Taq* DNA polymerase (New England Biolabs, MA, USA), and 2 µL DNA extract. Samples were amplified in triplicate under the following PCR conditions: 95°C for 3 min, 45 cycles of 95°C for 30 sec, 50°C for 30 sec, 68°C for 1 min, and a final extension step of 68°C for 7 min. The amplicons from triplicate reactions were quantified in an agarose gel, and equal quantities were pooled to a maximum volume of 20 µL. A volume of 5 µL was pooled for each control (no template and kit controls), even though no amplicons were detected. Pooled 16S rRNA gene amplicons were purified from the agarose gel using a Wizard SV Gel and PCR Clean-Up System (Promega, WI, USA) and quantified using a Qubit dsDNA High Sensitivity Assay Kit (Invitrogen, CA, USA). Sequencing was performed on a MiSeq System (Illumina, CA, USA) using a 2 × 250 cycle MiSeq Reagent Kit v2 (Illumina, NB, Canada) and 15% PhiX Control v3 (Illumina Canada).

### Sequence analysis

MiSeq Reporter software (version 2.5.0.5, Illumina) was used to demultiplex sequencing reads. Sequence reads were processed and analyzed using Quantitative Insights Into Microbial Ecology 2 (QIIME2; version 2023.5 [57]). Divisive Amplicon Denoising Algorithm (DADA2) (58) within QIIME 2023.5 was used to remove primer sequences and truncate forward and reverse reads after 250 and 239 bases, respectively. The DADA2 filtering, denoising, and chimera removal yielded a total of 4,758,430 sequences. Taxonomy was assigned to ASVs using a naive Bayes classifier (classify-sklearn) trained on SILVA version 138 (59). Bubble plots and PCoA plots were generated using the R-based visualization tool AOViz 2.4 (https://github.com/AlexUmbach/AOViz; R version 4.3.2). PCoA was performed using Bray-Curtis distances (60) and reads rarefied to 3,000 using ranked subsampling. Contaminant sequences based on negative controls (i.e., DNA extractions without sample added; PCR no template controls, kit controls) were identified using a decontam (61) prevalence method with a 0.5 score statistic threshold value and were removed from the ASV table.

### Natural organic matter analysis

Starting material and composites of inner layer samples from the two densities of year 7 modules were analyzed for total, inorganic, and organic carbon by University of Guelph, Laboratory Services. NOM chemistry was assessed via solid-state ^13^C NMR spectroscopy using a Bruker Avance III 500 MHz NMR spectrometer (Bruker BioSpin, Rheinstetten, Germany) and a 4 mm H-X MAS probe. NOM analysis details are provided in the SI Section II.

### Statistical analysis

Statistical analysis was performed using statistical software R, Excel, and the MetaboAnalyst online portal (62, 63). For all group comparisons (within and between PLFA, cultivation, and 16S rRNA gene data), ANOVA was used for analysis of normal and homogeneous data, whereas the Kruskal-Wallis and Dunn’s (performed in R version 4.3.1) tests were used for non-normal data. A *P* value < 0.05 was considered statistically significant. Where applicable, values are shown as average ± SD around the mean. For individual PLFA profiles in the samples, Spearman’s rank correlation was performed, and a correlation heat map was created by standardizing the PLFA data per sample as mole percentage. Significant difference between all sample groups in the PCoA ordination was assessed using the *qiime diversity adonis* function, and pairwise PERMANOVA was conducted using *qiime diversity beta-group-significance*. Faith’s phylogenetic diversity and phylotype richness were calculated using *qiime diversity alpha-phylogenetic* after rarefying all samples to 3,000 sequences per sample, and statistical difference was determined using the Kruskal-Wallis test.

## Data Availability

Sequences are available in the European Nucleotide Archive under accession number PRJEB76313.

## SUPPLEMENTAL MATERIAL

Supplemental material aem.01950-24-s0001.docx Figures S1 to S9; Tables S1 and S3 to S15.

Table S2 aem.01950-24-s0002.xlsx Spearman correlation P values.

ASM does not own the copyrights to Supplemental Material that may be linked to, or accessed through, an article. The authors have granted ASM a non-exclusive, world-wide license to publish the Supplemental Material files. Please contact the corresponding author directly for reuse.

## References

[1] Bengtsson A, Pedersen K. 2017. Microbial sulphide-producing activity in water saturated Wyoming MX-80, Asha and Calcigel bentonites at wet densities from 1500 to 2000kgm^−3^. Appl Clay Sci 137:203–212. doi:10.1016/j.clay.2016.12.024

[2] Crowe R, Birch K, Freire-Canosa J, Chen J, Doyle D, Garisto F, Gierszewski P, Gobien M, Boyle C, Hunt N, Hirschorn S, Jensen M, Keech P, Kennell-Morrison L, Kremer E, Medri C, Mielcarek M, Murchison A, Parmenter A, Sykes E, Yang T. 2017. Technical program for long-term management of Canada’s used nuclear fuel–annual report 2016. NWMO TR-2017-01. Nuclear Waste Management Organization, Toronto, Canada. https://www.nwmo.ca/-/media/Reports-MASTER/Technical-reports/NWMO-TR-2017-01-Technical-Program-for-Long-Term-Management-of-Canadas-Used-Nuclear-Fuel-2017-12.ashx?sc_lang=en.

[3] Hall DS, Behazin M, Jeffrey Binns W, Keech PG. 2021. An evaluation of corrosion processes affecting copper-coated nuclear waste containers in a deep geological repository. Prog Mater Sci 118:100766. doi:10.1016/j.pmatsci.2020.100766

[4] Masurat P, Eriksson S, Pedersen K. 2010. Microbial sulphide production in compacted Wyoming bentonite MX-80 under in situ conditions relevant to a repository for high-level radioactive waste. Appl Clay Sci 47:58–64. doi:10.1016/j.clay.2009.01.004

[5] Motamedi M, Karland O, Pedersen K. 1996. Survival of sulfate reducing bacteria at different water activities in compacted bentonite. FEMS Microbiol Lett 141:83–87. doi:10.1111/j.1574-6968.1996.tb08367.x

[6] Müller HR, Blechschmidt I, Vomvoris S, Vietor T, Alig M, Braun M. 2024. Status of the site investigation and site selection process for a deep geological repository in Switzerland. Nucl Technol 210:1740–1747. doi:10.1080/00295450.2023.2262298

[7] Pedersen K, Motamedi M, Karnland O, Sandén T. 2000. Cultivability of microorganisms introduced into a compacted bentonite clay buffer under high-level radioactive waste repository conditions. Eng Geol 58:149–161. doi:10.1016/S0013-7952(00)00056-9 11123477

[8] Ruiz-Fresneda MA, Martinez-Moreno MF, Povedano-Priego C, Morales-Hidalgo M, Jroundi F, Merroun ML. 2023. Impact of microbial processes on the safety of deep geological repositories for radioactive waste. Front Microbiol 14:1134078. doi:10.3389/fmicb.2023.1134078 37007474 PMC10062484

[9] Scully JR, Féron D, Hänninen H. 2016. Review of the NWMO copper corrosion allowance. TR-2016-11. Nuclear Waste Management Organization, Toronto, Canada. https://www.nwmo.ca/-/media/Reports-MASTER/Technical-reports/NWMO-TR-2016-11-Review-of-the-NWMO-Copper-Corrosion-Program-2016-08.ashx?sc_lang=en.

[10] Sellin P, Leupin OX. 2013. The use of clay as an engineered barrier in radioactive-waste management–a review. Clays and clay miner 61:477–498. doi:10.1346/CCMN.2013.0610601

[11] Stroes-Gascoyne S, Hamon CJ, Maak P, Russell S. 2010. The effects of the physical properties of highly compacted smectitic clay (bentonite) on the culturability of indigenous microorganisms. Appl Clay Sci 47:155–162. doi:10.1016/j.clay.2008.06.010

[12] Engel K, Ford SE, Binns WJ, Diomidis N, Slater GF, Neufeld JD. 2023. Stable microbial community in compacted bentonite after 5 years of exposure to natural granitic groundwater. mSphere 8:e0004823. doi:10.1128/msphere.00048-23 37772811 PMC10597416

[13] Leupin OX, Bernier-Latmani R, Bagnoud A, Moors H, Leys N, Wouters K, Stroes-Gascoyne S. 2017. Fifteen years of microbiological investigation in Opalinus Clay at the Mont Terri rock laboratory (Switzerland). Swiss J Geosci 110:343–354. doi:10.1007/s00015-016-0255-y PMC7081829 32214982

[14] Reddy B, Padovani C, Rance AP, Smart NR, Cook A, Haynes HM, Milodowski AE, Field LP, Kemp SJ, Martin A, Diomidis N. 2021. The anaerobic corrosion of candidate disposal canister materials in compacted bentonite exposed to natural granitic porewater containing native microbial populations. Mater Corros 72:361–382. doi:10.1002/maco.202011798

[15] Martinez-Moreno MF, Povedano-Priego C, Morales-Hidalgo M, Mumford AD, Ojeda JJ, Jroundi F, Merroun ML. 2023. Impact of compacted bentonite microbial community on the clay mineralogy and copper canister corrosion: a multidisciplinary approach in view of a safe Deep Geological Repository of nuclear wastes. J Hazard Mater 458:131940. doi:10.1016/j.jhazmat.2023.131940 37390682

[16] Jenni A, Wersin P, Thoenen T, Baeyens B, Ferrari A, Gimmi T, Mäder U, Marschall P, Hummel W, Leupin O. 2019. Bentonite backfill performance in a high-level waste repository: a geochemical perspective. Nagra Technical Report NTB 19-03. Available from: https://nagra.ch/en/downloads/technical-report-ntb-19-03-2/. Retrieved 10 Jan 2025.

[17] Chowdhury F, Rashwan TL, Mondal P, Behazin M, Keech PG, Sharma JS, Krol M. 2024. Effect of compaction on bisulfide diffusive transport through MX-80 bentonite. J Contam Hydrol 264:104341. doi:10.1016/j.jconhyd.2024.104341 38701693

[18] Papry SA, Rashwan TL, Mondal PK, Behazin M, Keech PG, Krol MM. 2023. Investigating bisulfide sorption onto bentonite through laboratory batch experiments. Appl Geochem 152:105626. doi:10.1016/j.apgeochem.2023.105626

[19] Engel K, Ford SE, Coyotzi S, McKelvie J, Diomidis N, Slater G, Neufeld JD. 2019. Stability of microbial community profiles associated with compacted bentonite from the Grimsel Underground Research Laboratory. mSphere 4:e00601-19. doi:10.1128/mSphere.00601-19 31852805 PMC6920512

[20] Moore-Kucera J, Dick RP. 2008. PLFA profiling of microbial community structure and seasonal shifts in soils of a Douglas-fir chronosequence. Microb Ecol 55:500–511. doi:10.1007/s00248-007-9295-1 17786504

[21] Kramer C, Gleixner G. 2006. Variable use of plant- and soil-derived carbon by microorganisms in agricultural soils. Soil Biol Biochem 38:3267–3278. doi:10.1016/j.soilbio.2006.04.006

[22] Grigoryan A, Stroes-Gascoyne S, Jalique DR, Wolfaardt GM, Keech P, McKelvie J, Korber DR. 2020. Baseline distribution and diversity of MIC-relevant microorganisms in compacted bentonites after incubation for 1 year. NACE - International Corrosion Conference Series; Houston

[23] Zelles L. 1997. Phospholipid fatty acid profiles in selected members of soil microbial communities. Chemosphere 35:275–294. doi:10.1016/s0045-6535(97)00155-0 9232001

[24] Usman MO, Simpson MJ. 2021. Assessment of the molecular-level compositional heterogeneity of natural organic matter in bentonites intended for long-term used nuclear fuel storage. Org Geochem 152:104166. doi:10.1016/j.orggeochem.2020.104166

[25] Burzan N, Murad Lima R, Frutschi M, Janowczyk A, Reddy B, Rance A, Diomidis N, Bernier-Latmani R. 2022. Growth and persistence of an aerobic microbial community in Wyoming Bentonite MX-80 despite anoxic in situ conditions. Front Microbiol 13:858324. doi:10.3389/fmicb.2022.858324 35547138 PMC9082992

[26] Schneeberger R, Kober F, Lanyon GW, Mäder UK, Spillmann T, Blechschmidt I. 2019. Grimsel test site: revisiting the site-specific geoscientific knowledge. Nagra Technical Report NTB 19-01. Available from: https://nagra.ch/en/downloads/technical-report-ntb-19-01-2/. Retrieved 10 Jan 2025.

[27] Allen LV. 2018. Quality control: water activity considerations for beyond-use dates. Int J Pharm Compd 22:288–293. 30021184

[28] Chi Fru E, Athar R. 2008. In situ bacterial colonization of compacted bentonite under deep geological high-level radioactive waste repository conditions. Appl Microbiol Biotechnol 79:499–510. doi:10.1007/s00253-008-1436-z 18379777

[29] Jalique DR, Stroes-Gascoyne S, Hamon CJ, Priyanto DG, Kohle C, Evenden WG, Wolfaardt GM, Grigoryan AA, McKelvie J, Korber DR. 2016. Culturability and diversity of microorganisms recovered from an eight-year old highly-compacted, saturated MX-80 Wyoming bentonite plug. Appl Clay Sci 126:245–250. doi:10.1016/j.clay.2016.03.022

[30] Persson J, Lydmark S, Edlund J, Pääjärvi A, Karsten P. 2011. Microbial incidence on copper and titanium embedded in compacted bentonite clay. Report R-11-22. Swedish nuclear fuel and waste management Company, Stockholm, Sweden. https://inis.iaea.org/collection/NCLCollectionStore/_Public/43/084/43084408.pdf?r=1.

[31] Stroes-Gascoyne S, Pedersen K, Haveman SA, Dekeyser K, Arlinger J, Daumas S, Ekendahl S, Hallbeck L, Hamon CJ, Jahromi N, Delaney TL. 1997. Occurrence and identification of microorganisms in compacted clay-based buffer material designed for use in a nuclear fuel waste disposal vault. Can J Microbiol 43:1133–1146. doi:10.1139/m97-162 9476350

[32] Phaiboun A, Zhang Y, Park B, Kim M. 2015. Survival kinetics of starving bacteria is biphasic and density-dependent. PLoS Comput Biol 11:e1004198. doi:10.1371/journal.pcbi.1004198 25838110 PMC4383377

[33] Shoemaker WR, Jones SE, Muscarella ME, Behringer MG, Lehmkuhl BK, Lennon JT. 2021. Microbial population dynamics and evolutionary outcomes under extreme energy limitation. Proc Natl Acad Sci U S A 118:e2101691118. doi:10.1073/pnas.2101691118 34385301 PMC8379937

[34] Frostegård Å, Tunlid A, Bååth E. 2011. Use and misuse of PLFA measurements in soils. Soil Biol Biochem 43:1621–1625. doi:10.1016/j.soilbio.2010.11.021

[35] Pinkart HC, Ringelberg DB, Piceno YM, MacNaughton SJ, White DC. 2002. Biochemical approaches to biomass measurements and community structure analysis, p 101–113. In Hurst CJ, Crawford RL, Knudsen GR, McInerney MJ, Stetzenbach LD (ed), Manual of environmental microbiology, 2nd ed. ASM Press, Washington DC.

[36] Vestal JR. 1988. Biomass of the cryptoendolithic microbiota from the Antarctic desert. Appl Environ Microbiol 54:957–959. doi:10.1128/aem.54.4.957-959.1988 11536603 PMC202579

[37] White DC, Tucker AN. 1969. Phospholipid metabolism during bacterial growth. J Lipid Res 10:220–233. 4305713

[38] Lovley DR, Chapelle FH. 1995. Deep subsurface microbial processes. Rev Geophys 33:365–381. doi:10.1029/95RG01305

[39] Boivin-Jahns V, Ruimy R, Bianchi A, Daumas S, Christen R. 1996. Bacterial diversity in a deep-subsurface clay environment. Appl Environ Microbiol 62:3405–3412. doi:10.1128/aem.62.9.3405-3412.1996 8795233 PMC168139

[40] Suzuki Y, Webb SJ, Kouduka M, Kobayashi H, Castillo J, Kallmeyer J, Moganedi K, Allwright AJ, Klemd R, Roelofse F, Mapiloko M, Hill SJ, Ashwal LD, Trumbull RB. 2024. Subsurface microbial colonization at mineral-filled veins in 2-billion-year-old Mafic Rock from the Bushveld Igneous Complex, South Africa. Microb Ecol 87:116. doi:10.1007/s00248-024-02434-8 39354222 PMC11445344

[41] Fredrickson JK, Mckinley JP, Nierzwicki‐bauer SA, White DC, Ringelberg DB, Rawson SA, Li S, Brockman FJ, Bjornstad BN. 1995. Microbial community structure and biogeochemistry of Miocene subsurface sediments: implications for long‐term microbial survival. Mol Ecol 4:619–626. doi:10.1111/j.1365-294X.1995.tb00262.x

[42] Phelps TJ, Murphy EM, Pfiffner SM, White DC. 1994. Comparison between geochemical and biological estimates of subsurface microbial activities. Microb Ecol 28:335–349. doi:10.1007/BF00662027 24186553

[43] Jørgensen BB. 2011. Deep subseafloor microbial cells on physiological standby. Proc Natl Acad Sci U S A 108:18193–18194. doi:10.1073/pnas.1115421108 22031692 PMC3215010

[44] Rempfert KR, Miller HM, Bompard N, Nothaft D, Matter JM, Kelemen P, Fierer N, Templeton AS. 2017. Geological and geochemical controls on subsurface microbial life in the Samail Ophiolite, Oman. Front Microbiol 8:56. doi:10.3389/fmicb.2017.00056 28223966 PMC5293757

[45] Stroes-Gascoyne S, Hamon CJ, Maak P. 2011. Limits to the use of highly compacted bentonite as a deterrent for microbiologically influenced corrosion in a nuclear fuel waste repository. Phys Chem Earth 36:1630–1638. doi:10.1016/j.pce.2011.07.085

[46] Pedersen K. 2010. Analysis of copper corrosion in compacted bentonite clay as a function of clay density and growth conditions for sulfate-reducing bacteria. J Appl Microbiol 108:1094–1104. doi:10.1111/j.1365-2672.2009.04629.x 20015208

[47] Briggs S, McKelvie J, Sleep B, Krol M. 2017. Multi-dimensional transport modelling of corrosive agents through a bentonite buffer in a Canadian deep geological repository. Sci Total Environ 599–600:348–354. doi:10.1016/j.scitotenv.2017.04.242 28478364

[48] Maanoja S, Palmroth M, Salminen L, Lehtinen L, Kokko M, Lakaniemi A-M, Auvinen H, Kiczka M, Muuri E, Rintala J. 2021. The effect of compaction and microbial activity on the quantity and release rate of water-soluble organic matter from bentonites. Appl Clay Sci 211:106192. doi:10.1016/j.clay.2021.106192

[49] Karnland O, Olsson S, Nilsson U. 2006. Mineralogy and sealing properties of various bentonites and smectite-rich clay materials. Technical Report TR-06-30. Clay Technol AB, Stockholm. https://skb.com/publication/1419144.

[50] Bligh EG, Dyer WJ. 1959. A rapid method of total lipid extraction and purification. Can J Biochem Physiol 37:911–917. doi:10.1139/o59-099 13671378

[51] White DC, Ringelberg DB. 1998. Signature lipid biomarker analysis, p 255–272. In Burlage RS, Atlas R, Stahl D, Geesey G, Sayler G (ed), Techniques in microbial ecology. Oxford University Press, New York.

[52] Green CT, Scow KM. 2000. Analysis of phospholipid fatty acids (PLFA) to characterize microbial communities in aquifers. Hydrogeol J 8:126–141. doi:10.1007/s100400050013

[53] Muyzer G, de Waal EC, Uitterlinden AG. 1993. Profiling of complex microbial populations by denaturing gradient gel electrophoresis analysis of polymerase chain reaction-amplified genes coding for 16S rRNA. Appl Environ Microbiol 59:695–700. doi:10.1128/aem.59.3.695-700.1993 7683183 PMC202176

[54] Beaver RC, Vachon MA, Tully CS, Engel K, Spasov E, Binns WJ, Noël JJ, Neufeld JD. 2024. Impact of dry density and incomplete saturation on microbial growth in bentonite clay for nuclear waste storage. J Appl Microbiol 135:lxae053. doi:10.1093/jambio/lxae053 38458234

[55] Parada AE, Needham DM, Fuhrman JA. 2016. Every base matters: assessing small subunit rRNA primers for marine microbiomes with mock communities, time series and global field samples. Environ Microbiol 18:1403–1414. doi:10.1111/1462-2920.13023 26271760

[56] Quince C, Lanzen A, Davenport RJ, Turnbaugh PJ. 2011. Removing noise from pyrosequenced amplicons. BMC Bioinformatics 12:38. doi:10.1186/1471-2105-12-38 21276213 PMC3045300

[57] Bolyen E, Rideout JR, Dillon MR, Bokulich NA, Abnet CC, Al-Ghalith GA, Alexander H, Alm EJ, Arumugam M, Asnicar F, et al.. 2019. Reproducible, interactive, scalable and extensible microbiome data science using QIIME 2. Nat Biotechnol 37:852–857. doi:10.1038/s41587-019-0209-9 31341288 PMC7015180

[58] Callahan BJ, McMurdie PJ, Rosen MJ, Han AW, Johnson AJA, Holmes SP. 2016. DADA2: High-resolution sample inference from Illumina amplicon data. Nat Methods 13:581–583. doi:10.1038/nmeth.3869 27214047 PMC4927377

[59] Quast C, Pruesse E, Yilmaz P, Gerken J, Schweer T, Yarza P, Peplies J, Glöckner FO. 2013. The SILVA ribosomal RNA gene database project: improved data processing and web-based tools. Nucleic Acids Res 41:D590–D596. doi:10.1093/nar/gks1219 23193283 PMC3531112

[60] Bray JR, Curtis JT. 1957. An ordination of the upland forest communities of southern Wisconsin. Ecol Monogr 27:325–349. doi:10.2307/1942268

[61] Davis NM, Proctor DM, Holmes SP, Relman DA, Callahan BJ. 2018. Simple statistical identification and removal of contaminant sequences in marker-gene and metagenomics data. Microbiome 6:226. doi:10.1186/s40168-018-0605-2 30558668 PMC6298009

[62] Pang Z, Chong J, Zhou G, de Lima Morais DA, Chang L, Barrette M, Gauthier C, Jacques P-É, Li S, Xia J. 2021. MetaboAnalyst 5.0: narrowing the gap between raw spectra and functional insights. Nucleic Acids Res 49:W388–W396. doi:10.1093/nar/gkab382 34019663 PMC8265181

[63] Xia J, Psychogios N, Young N, Wishart DS. 2009. MetaboAnalyst: a web server for metabolomic data analysis and interpretation. Nucleic Acids Res 37:W652–W660. doi:10.1093/nar/gkp356 19429898 PMC2703878

