# Supplementary material for: Minimal changes in microbial abundances and diversity over 7 years of emplacement for modules of compacted bentonite exposed to natural groundwater
Source: Appl Environ Microbiol. 2025 Feb 25;91(3):e01950-24. doi: 10.1128/aem.01950-24 (PMC11921334; doi:10.1128/aem.01950-24)
Supplement: Supplemental material — Figures S1 to S9; Tables S1 and S3 to S15. [file aem.01950-24-s0001.docx]

**Minimal changes in microbial abundances and diversity over 7 years of emplacement for modules of compacted bentonite exposed to natural groundwater**

Harmanpreet Sidhu^a^, Katja Engel^b^, Sian E. Ford^a^, Peter Keech^c^, Mehran Behazin^c^, W.Jeffrey Binns^c^ Nivetha Srikanthan^d^, Myrna J. Simpson^d^, Josh D. Neufeld^b^, and Gregory F. Slater^a^

^a^School of Earth, Environment & Society, McMaster University, Hamilton, ON, L8S 4L8, Canada

^b^Department of Biology, University of Waterloo, Waterloo, ON, N2L 3G1, Canada

^c^Nuclear Waste Management Organization, Toronto, ON, M4T 2S3, Canada

^d^Department of Physical & Environmental Sciences, University of Toronto Scarborough, Toronto, ON, M1C 1A4, Canada

Running Head: Microbial changes in compacted bentonite over 7 years

***Supporting Information***

**Section I. PLFA abundance and profile data**

**Table S1.** Total PLFA abundances and number of PLFAs detected (± SD) in the starting material and the inner and outer layers of the bentonite modules at the dry densities of 1.25 g cm^-3^ or 1.50 g cm^-3^ retrieved at years 1, 5, and 7.

| Sample Information | | | Number of replicates | Number of PLFA detected | Total PLFA Abundance (pmol g^-1^) |
| --- | --- | --- | --- | --- | --- |
| Starting material | Year 0 | MX6 | 2 | 23 ± 10 | 35 ± 9.2 |
|  |  | MX7 | 2 | 26 ± 8 | 93 ± 35 |
|  |  | MX6-7* average | 4 | 24 ± 7 | 64 ± 39 |
| 1.25 g cm^-3^ | Year 1 | Inner layer | 4 | 22 ± 11 | 230 ± 220 |
|  |  | Outer layer | 3 | 24 ± 9 | 360 ± 210 |
|  | Year 5 | Inner layer | 2 | 5 ± 0 | 93 ± 39 |
|  |  | Outer layer | 2 | 24 ± 1 | 520 ± 46 |
|  | Year 7 | Inner layer | 4 | 34 ± 7 | 250 ± 290 |
|  |  | Outer layer | 5 | 30 ± 10 | 850 ± 320 |
| 1.50 g cm^-3^ | Year 1 | Inner layer | 4 | 17 ± 9 | 140 ± 96 |
|  |  | Outer layer | 3 | 16 ± 6 | 210 ± 150 |
|  | Year 5 | Inner layer | 2 | 34 ± 4 | 130 ± 3.7 |
|  |  | Outer layer | 2 | 29 ± 4 | 510 ± 20 |
|  | Year 7 | Inner layer | 5 | 36 ± 4 | 210 ± 130 |
|  |  | Outer layer | 4 | 27 ± 10 | 750 ± 370 |

*Two replicates of the starting material (one each of MX6 and MX7) were analyzed with year 5 samples and two were analyzed with year 7 samples (total 4 replicates).


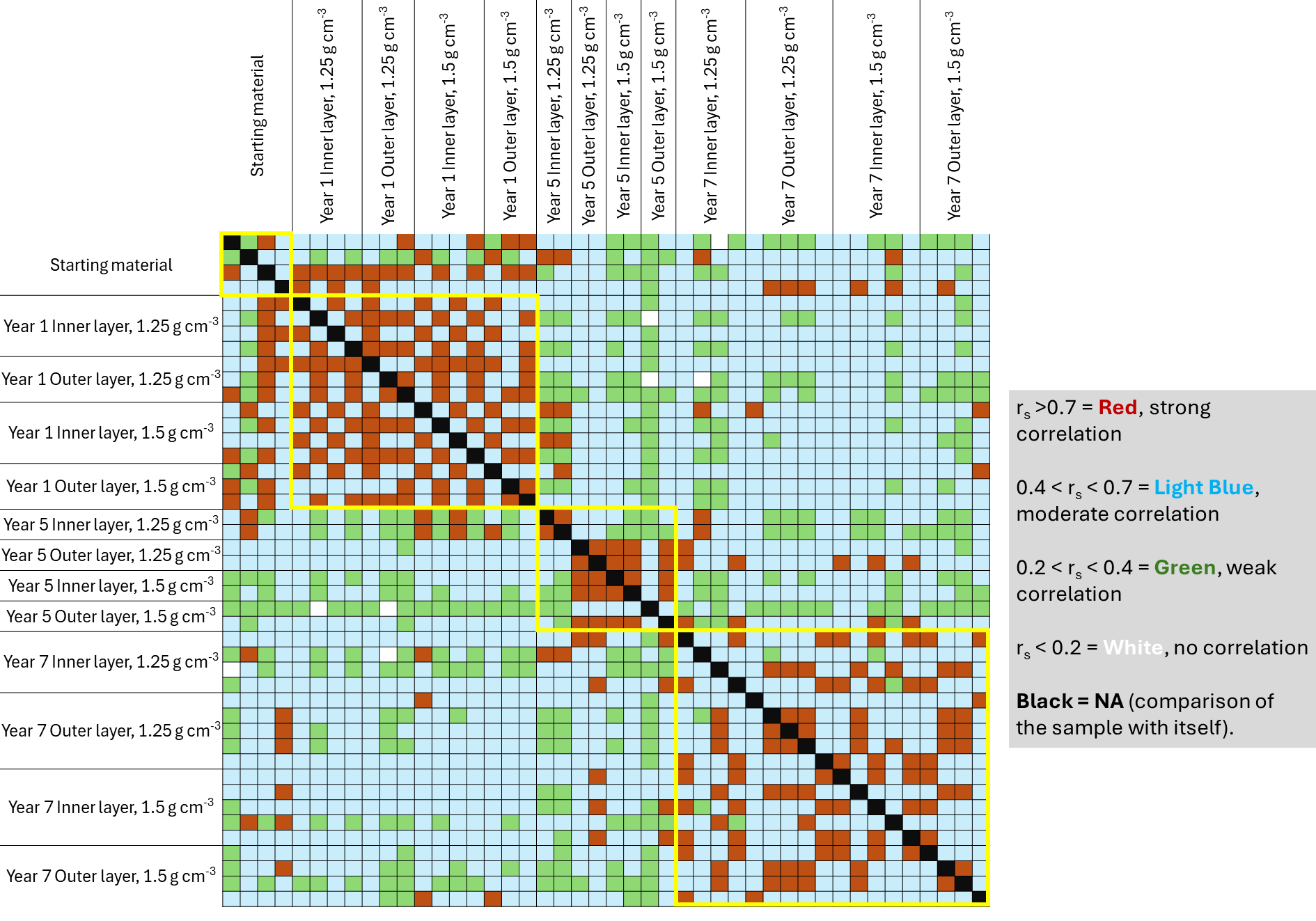


**Figure S1.** Spearman correlation analysis-based heatmap of all pairwise relationships among PLFAs obtained from samples analyzed across the 7-year deployment of MaCoTe modules. Red blocks indicate a strong positive correlation (r_s_ > 0.7), light blue a moderate positive correlation (0.4 < r_s_ < 0.7), green a weak positive correlation (0.2 < r_s_ < 0.4), and white no correlation (r_s_ < 0.2). Black blocks indicate a perfect correlation (r_s_ = 1); correlation of a sample with itself). Details regarding *p* values can be found in Table S2. The yellow rectangles highlight temporal clustering (with relatively greater correlations) of the samples, suggesting minute potential microbial PLFA profile changes over the years.

**Table S2. See Excel file**

**Table S3.** Results of Dunn’s test with Benjamini-Hochberg *p* value adjustment for PLFA profile comparisons in outer and inner layers of borehole samples from years 0 (starting material), 1, 5, and 7. Significant differences below α of 0.01 are indicated by ** and between 0.01 and 0.05 are indicated by *.

| Group 1 | Group 2 | Z | P.unadj. | P.adj. | Significance |
| --- | --- | --- | --- | --- | --- |
| Starting material | Year 1 inner layer bentonite 1.25 | 0.6043 | 0.5457 | 0.644918 |  |
| Starting material | Year 1 outer layer bentonite 1.25 | 0.03956 | 0.9684 | 0.980977 |  |
| Starting material | Year 5 inner layer bentonite 1.25 | 4.1572 | 3.22E-05 | 0.000193 | ** |
| Starting material | Year 5 outer layer bentonite 1.25 | 1.0133 | 0.3109 | 0.41102 |  |
| Starting material | Year 7 inner layer bentonite 1.25 | 3.2761 | 0.001052 | 0.003156 | ** |
| Starting material | Year 7 outer layer bentonite 1.25 | 2.2418 | 0.02498 | 0.050193 |  |
| Starting material | Year 1 inner layer bentonite 1.50 | 1.7914 | 0.07322 | 0.124156 |  |
| Starting material | Year 1 outer layer bentonite 1.50 | 1.7009 | 0.08896 | 0.147636 |  |
| Starting material | Year 5 inner layer bentonite 1.50 | 2.319 | 0.02039 | 0.042984 | * |
| Starting material | Year 5 outer layer bentonite 1.50 | 1.9045 | 0.05685 | 0.103123 |  |
| Starting material | Year 7 inner layer bentonite 1.50 | 3.5817 | 0.000341 | 0.001158 | ** |
| Starting material | Year 7 outer layer bentonite 1.50 | 2.2334 | 0.02552 | 0.050193 |  |
| Year 1 inner layer bentonite 1.25 | Year 1 outer layer bentonite 1.25 | 0.5199 | 0.6032 | 0.702233 |  |
| Year 1 inner layer bentonite 1.25 | Year 5 inner layer bentonite 1.25 | 3.6638 | 0.000249 | 0.000923 | ** |
| Year 1 inner layer bentonite 1.25 | Year 5 outer layer bentonite 1.25 | 1.5067 | 0.1319 | 0.201729 |  |
| Year 1 inner layer bentonite 1.25 | Year 7 inner layer bentonite 1.25 | 3.8804 | 0.000104 | 0.000479 | ** |
| Year 1 inner layer bentonite 1.25 | Year 7 outer layer bentonite 1.25 | 2.846 | 0.004427 | 0.011814 | * |
| Year 1 inner layer bentonite 1.25 | Year 1 inner layer bentonite 1.50 | 1.1872 | 0.2352 | 0.339733 |  |
| Year 1 inner layer bentonite 1.25 | Year 1 outer layer bentonite 1.50 | 1.1415 | 0.2537 | 0.359496 |  |
| Year 1 inner layer bentonite 1.25 | Year 5 inner layer bentonite 1.50 | 2.8124 | 0.004917 | 0.012372 | * |
| Year 1 inner layer bentonite 1.25 | Year 5 outer layer bentonite 1.50 | 2.3979 | 0.01649 | 0.036749 | * |
| Year 1 inner layer bentonite 1.25 | Year 7 inner layer bentonite 1.50 | 4.2186 | 2.46E-05 | 0.00016 | ** |
| Year 1 inner layer bentonite 1.25 | Year 7 outer layer bentonite 1.50 | 2.8377 | 0.004544 | 0.011814 | * |
| Year 1 outer layer bentonite 1.25 | Year 5 inner layer bentonite 1.25 | 3.9108 | 0.000092 | 0.000449 | ** |
| Year 1 outer layer bentonite 1.25 | Year 5 outer layer bentonite 1.25 | 0.9944 | 0.32 | 0.416 |  |
| Year 1 outer layer bentonite 1.25 | Year 7 inner layer bentonite 1.25 | 3.0727 | 0.002122 | 0.005911 | ** |
| Year 1 outer layer bentonite 1.25 | Year 7 outer layer bentonite 1.25 | 2.115 | 0.03443 | 0.065167 |  |
| Year 1 outer layer bentonite 1.25 | Year 1 inner layer bentonite 1.50 | 1.619 | 0.1054 | 0.16778 |  |
| Year 1 outer layer bentonite 1.25 | Year 1 outer layer bentonite 1.50 | 1.5541 | 0.1202 | 0.187512 |  |
| Year 1 outer layer bentonite 1.25 | Year 5 inner layer bentonite 1.50 | 2.2331 | 0.02574 | 0.050193 |  |
| Year 1 outer layer bentonite 1.25 | Year 5 outer layer bentonite 1.50 | 1.8398 | 0.06579 | 0.116628 |  |
| Year 1 outer layer bentonite 1.25 | Year 7 inner layer bentonite 1.50 | 3.3313 | 0.000864 | 0.002697 | ** |
| Year 1 outer layer bentonite 1.25 | Year 7 outer layer bentonite 1.50 | 2.1073 | 0.03509 | 0.065167 |  |
| Year 5 inner layer bentonite 1.25 | Year 5 outer layer bentonite 1.25 | 4.4778 | 7.54E-06 | 5.35E-05 | ** |
| Year 5 inner layer bentonite 1.25 | Year 7 inner layer bentonite 1.25 | 6.8321 | 8.37E-12 | 3.26E-10 | ** |
| Year 5 inner layer bentonite 1.25 | Year 7 outer layer bentonite 1.25 | 5.9876 | 2.13E-09 | 4.33E-08 | ** |
| Year 5 inner layer bentonite 1.25 | Year 1 inner layer bentonite 1.50 | 2.6945 | 0.00705 | 0.017184 | * |
| Year 5 inner layer bentonite 1.25 | Year 1 outer layer bentonite 1.50 | 2.5208 | 0.01171 | 0.027678 | * |
| Year 5 inner layer bentonite 1.25 | Year 5 inner layer bentonite 1.50 | 5.6086 | 2.04E-08 | 3.18E-07 | ** |
| Year 5 inner layer bentonite 1.25 | Year 5 outer layer bentonite 1.50 | 5.2496 | 1.53E-07 | 1.7E-06 | ** |
| Year 5 inner layer bentonite 1.25 | Year 7 inner layer bentonite 1.50 | 7.1748 | 7.24E-13 | 5.65E-11 | ** |
| Year 5 inner layer bentonite 1.25 | Year 7 outer layer bentonite 1.50 | 5.9808 | 2.22E-09 | 4.33E-08 | ** |
| Year 5 outer layer bentonite 1.25 | Year 7 inner layer bentonite 1.25 | 1.6616 | 0.09658 | 0.156943 |  |
| Year 5 outer layer bentonite 1.25 | Year 7 outer layer bentonite 1.25 | 0.8171 | 0.4139 | 0.517276 |  |
| Year 5 outer layer bentonite 1.25 | Year 1 inner layer bentonite 1.50 | 2.476 | 0.01329 | 0.030489 | * |
| Year 5 outer layer bentonite 1.25 | Year 1 outer layer bentonite 1.50 | 2.3844 | 0.01711 | 0.037072 | * |
| Year 5 outer layer bentonite 1.25 | Year 5 inner layer bentonite 1.50 | 1.1308 | 0.2581 | 0.359496 |  |
| Year 5 outer layer bentonite 1.25 | Year 5 outer layer bentonite 1.50 | 0.7718 | 0.4402 | 0.5292 |  |
| Year 5 outer layer bentonite 1.25 | Year 7 inner layer bentonite 1.50 | 1.8229 | 0.06833 | 0.118439 |  |
| Year 5 outer layer bentonite 1.25 | Year 7 outer layer bentonite 1.50 | 0.8103 | 0.4178 | 0.517276 |  |
| Year 7 inner layer bentonite 1.25 | Year 7 outer layer bentonite 1.25 | 1.0343 | 0.301 | 0.404793 |  |
| Year 7 inner layer bentonite 1.25 | Year 1 inner layer bentonite 1.50 | 5.0676 | 4.03E-07 | 3.49E-06 | ** |
| Year 7 inner layer bentonite 1.25 | Year 1 outer layer bentonite 1.50 | 4.734 | 2.2E-06 | 1.72E-05 | ** |
| Year 7 inner layer bentonite 1.25 | Year 5 inner layer bentonite 1.50 | 0.3559 | 0.7219 | 0.782058 |  |
| Year 7 inner layer bentonite 1.25 | Year 5 outer layer bentonite 1.50 | 0.7705 | 0.441 | 0.5292 |  |
| Year 7 inner layer bentonite 1.25 | Year 7 inner layer bentonite 1.50 | 0.1283 | 0.8979 | 0.9594 |  |
| Year 7 inner layer bentonite 1.25 | Year 7 outer layer bentonite 1.50 | 1.0427 | 0.2971 | 0.404793 |  |
| Year 7 outer layer bentonite 1.25 | Year 1 inner layer bentonite 1.50 | 4.0332 | 5.5E-05 | 0.000296 | ** |
| Year 7 outer layer bentonite 1.25 | Year 1 outer layer bentonite 1.50 | 3.7764 | 0.000159 | 0.00064 | ** |
| Year 7 outer layer bentonite 1.25 | Year 5 inner layer bentonite 1.50 | 0.4886 | 0.6251 | 0.706635 |  |
| Year 7 outer layer bentonite 1.25 | Year 5 outer layer bentonite 1.50 | 0.07408 | 0.941 | 0.97864 |  |
| Year 7 outer layer bentonite 1.25 | Year 7 inner layer bentonite 1.50 | 1.2186 | 0.223 | 0.328189 |  |
| Year 7 outer layer bentonite 1.25 | Year 7 outer layer bentonite 1.50 | 0.008359 | 0.9933 | 0.9933 |  |
| Year 1 inner layer bentonite 1.50 | Year 1 outer layer bentonite 1.50 | 0.04236 | 0.9662 | 0.980977 |  |
| Year 1 inner layer bentonite 1.50 | Year 5 inner layer bentonite 1.50 | 3.7817 | 0.000156 | 0.00064 | ** |
| Year 1 inner layer bentonite 1.50 | Year 5 outer layer bentonite 1.50 | 3.3672 | 0.000759 | 0.002468 | ** |
| Year 1 inner layer bentonite 1.50 | Year 7 inner layer bentonite 1.50 | 5.47 | 4.5E-08 | 5.85E-07 | ** |
| Year 1 inner layer bentonite 1.50 | Year 7 outer layer bentonite 1.50 | 4.0249 | 5.7E-05 | 0.000296 | ** |
| Year 1 outer layer bentonite 1.50 | Year 5 inner layer bentonite 1.50 | 3.6231 | 0.000291 | 0.001032 | ** |
| Year 1 outer layer bentonite 1.50 | Year 5 outer layer bentonite 1.50 | 3.2298 | 0.001239 | 0.003579 | ** |
| Year 1 outer layer bentonite 1.50 | Year 7 inner layer bentonite 1.50 | 5.0688 | 4E-07 | 3.49E-06 | ** |
| Year 1 outer layer bentonite 1.50 | Year 7 outer layer bentonite 1.50 | 3.7687 | 0.000164 | 0.00064 | ** |
| Year 5 inner layer bentonite 1.50 | Year 5 outer layer bentonite 1.50 | 0.359 | 0.7196 | 0.782058 |  |
| Year 5 inner layer bentonite 1.50 | Year 7 inner layer bentonite 1.50 | 0.4713 | 0.6374 | 0.710246 |  |
| Year 5 inner layer bentonite 1.50 | Year 7 outer layer bentonite 1.50 | 0.4954 | 0.6203 | 0.706635 |  |
| Year 5 outer layer bentonite 1.50 | Year 7 inner layer bentonite 1.50 | 0.9004 | 0.3679 | 0.47043 |  |
| Year 5 outer layer bentonite 1.50 | Year 7 outer layer bentonite 1.50 | 0.0809 | 0.9355 | 0.97864 |  |
| Year 7 inner layer bentonite 1.50 | Year 7 outer layer bentonite 1.50 | 1.2274 | 0.2197 | 0.328189 |  |

**Section II. Analysis of Natural Organic Matter Chemistry in MaCoTe Module year 7 samples**

**Materials and Methods**

*Sample preparation*: Sub-samples from sections 1 to 5 of the two density bentonite module samples were of insufficient mass to carry out complete natural organic matter characterization. As such, composite samples were prepared by combining samples from sections 1 and 5 together (composite referred to as “S1/S5 composite”) and combining samples from sections 2, 3, and 4 together (composite referred to as “S2/S3/S4 composite”). Samples were received frozen and were freeze-dried to remove water before preparing composite samples of equal masses from the different sections. Composite samples were kept frozen prior to further analyses. Subsamples of the bentonite used to prepare the modules were also obtained and freeze-dried prior to analysis (referred to as the reference bentonites).

*Total Carbon, Inorganic Carbon, and Organic Carbon Analysis*

Total carbon, inorganic carbon, and organic carbon were determined by the University of Guelph, Laboratory Services. Approximately 2 grams of freeze-dried and ground composite and reference bentonite samples were submitted for analyses. Measurements were conducted using a LECO CN828 carbon analyzer which quantified total carbon and inorganic carbon. Organic carbon was determined by difference.

*Solid-state ^13^C Nuclear Magnetic Resonance (NMR) Spectroscopy*

Composite and reference samples were repeatedly extracted with a mixture of hydrofluoric acid (10% v/v) and hydrochloric acid (4% v/v) to dissolve minerals and enrich carbon found in natural organic matter. After mineral dissolution, residues were repeatedly rinsed with deionized water to remove excess ions and then freeze-dried for NMR analysis.

Solid-state ^13^C NMR spectra were acquired using a Bruker Avance III 500 MHz NMR spectrometer (Bruker BioSpin, Rheinstetten, Germany) and a 4 mm H-X MAS probe. Samples were packed into 4mm zirconium rotors, sealed with a Kel-F cap. Ramp-cross polarization, a magic angle spinning rate of 11 kHz, a cross polarization contact time of 1 ms and 1 s recycle delay were used (1-3). NMR spectra were processed using with Bruker TopSpin (version 3.6.2) using a line broadening of 100 Hz and calibrated against a glycine standard. Spectra were integrated into four regions: alkyl (0-50 ppm), *O*-alkyl (50-110 ppm), aromatic and phenolic (110 – 165 ppm), and carboxylic and carbonyl (165- 230 ppm) carbon.

**Results and Discussion**

*Solid-state ^13^C Nuclear Magnetic Resonance (NMR) Spectroscopy*

Solid-state ^13^C NMR spectra provide an overview of all natural organic matter components. A comparison of the two reference samples (Figure S2; Table S4) shows that the natural organic matter was dominated by alkyl and aromatic carbon, which is similar to other studies that have examined Wyoming type bentonites (MX-80) (1, 2, 4). This chemistry is consistent with diagenetic alteration of natural organic matter which limits its reactivity. However, it is also established that there is some natural organic matter variability (up to 6% as detected by NMR) (1) so we also compared a mixture of both reference materials (Figure S2; Table S4) and found low heterogeneity in the chemistry of the natural organic matter which agrees with our previous solid-state ^13^C NMR analyses of bentonite clays.

A comparison of the outer and inner layer composites for both densities (1.25 and 1.50 g cm^-3^) to the reference materials (Table S4; Figure S3) does not reveal any major differences in the composition of natural organic matter with compaction. This agrees with other studies that found that the natural organic matter in Wyoming type bentonite clays is chemically stable with compaction (5), heat exposure (2), radiation exposure (3), and salinity (with and without heat exposure) (3). Taken together, the natural organic matter does not appear to have been altered in the MaCoTe modules beyond the level related to the inherent natural variability.


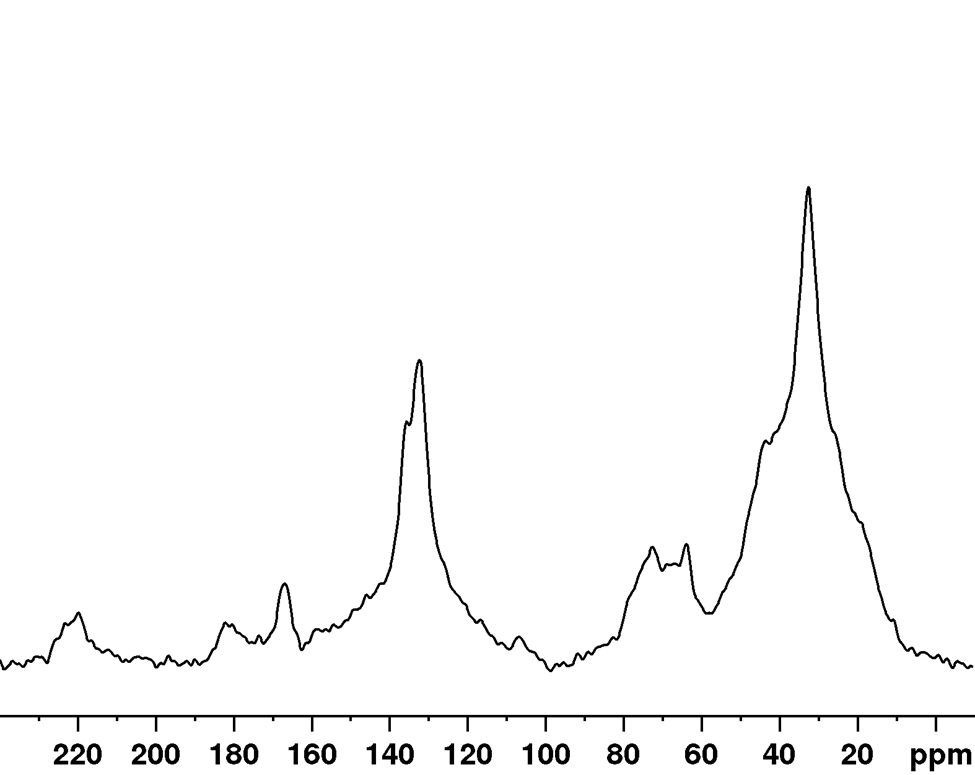


Reference – fine (MX6)

Reference – coarse (MX7)

Mix of reference materials


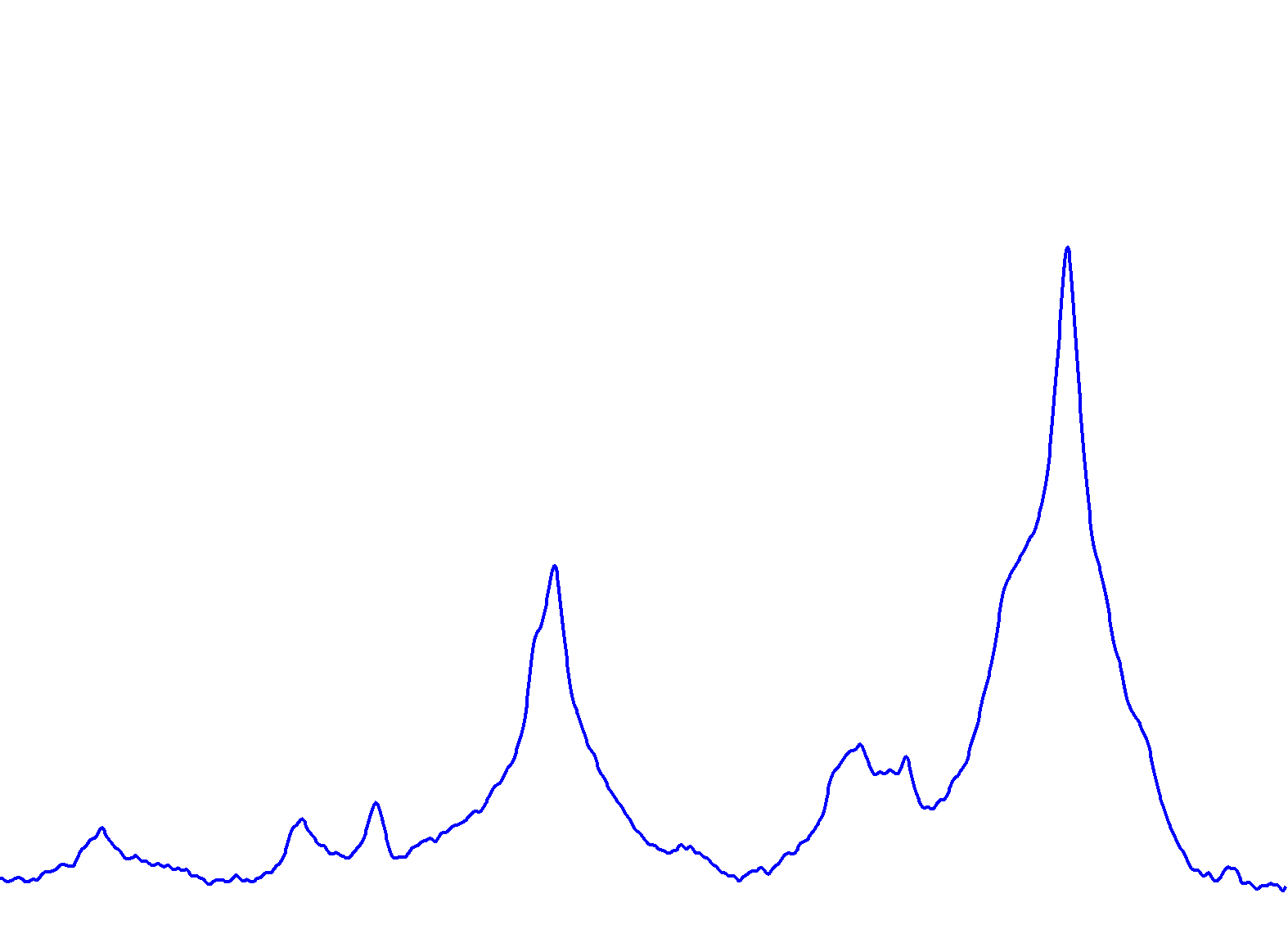

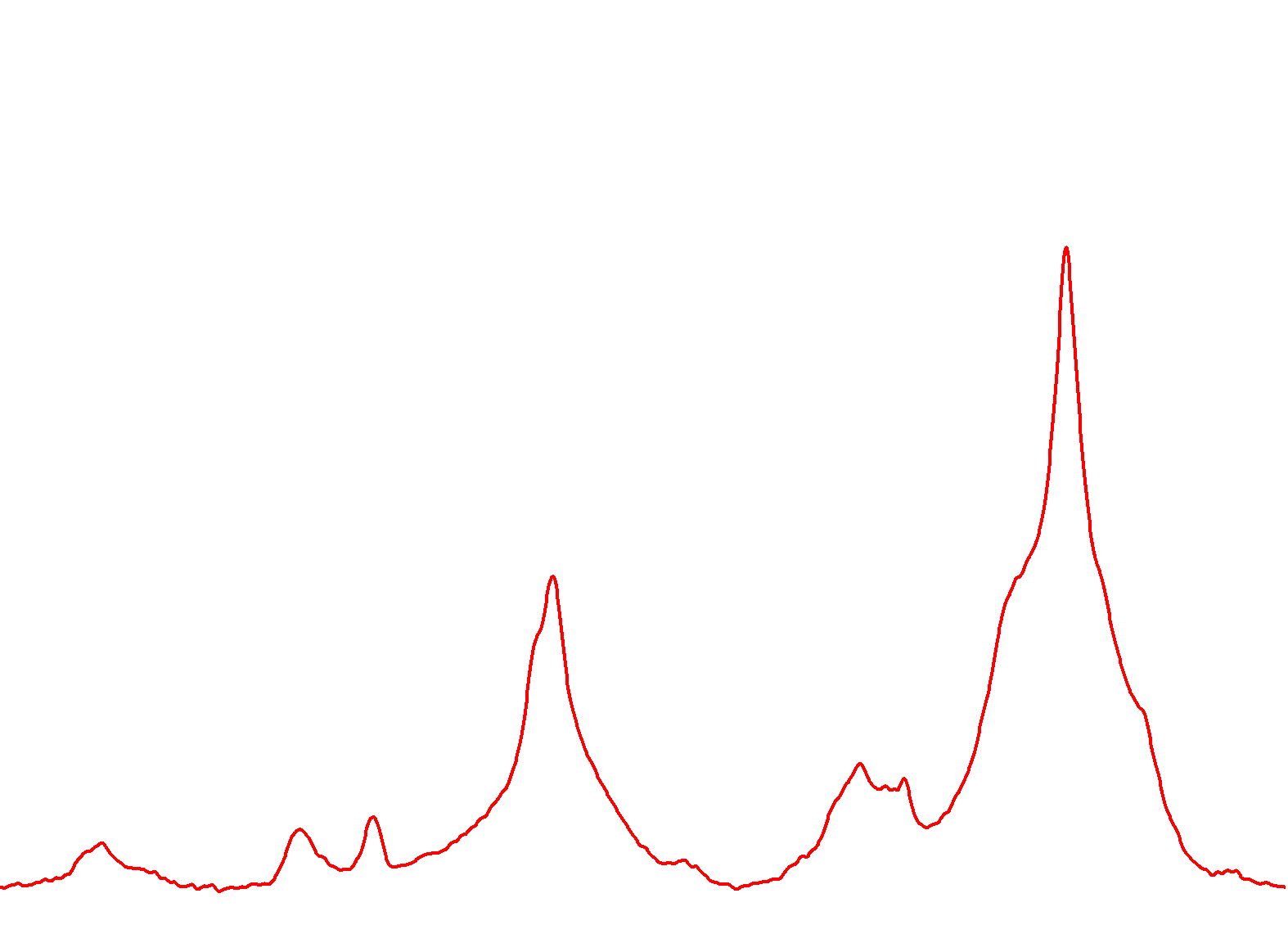


**^13^C Chemical Shift**

**Figure S2:** Solid-state ^13^C NMR spectra of reference samples and a mixture of both fine and coarse bentonites used to create the MaCoTe modules. The mixture of both reference materials was prepared by combining an equal mass of each sample.

**Table S4:** Solid-state ^13^C integration values (% of total signal) for the main categories of natural organic matter.

| **Sample Name** | **Alkyl carbon**  **(0-50 ppm)** | ***O*-alkyl carbon**  **(50-110 ppm)** | **Aromatic + phenolic carbon (110-165 ppm)** | **Carboxylic + carbonyl carbon**  **(165 – 230 ppm)** | **Alkyl/*O*-alkyl carbon^1^** |
| --- | --- | --- | --- | --- | --- |
| Reference - Fine | 48 | 17 | 27 | 8 | 2.82 |
| Reference - Coarse | 50 | 18 | 25 | 7 | 2.78 |
| Reference – mix | 53 | 15 | 25 | 7 | 3.53 |
| S1/S5 composite - 1.25 g cm^-3^ | 53 | 15 | 24 | 8 | 3.53 |
| S2/S3/S4 composite - 1.25 g cm^-3^ | 54 | 15 | 24 | 7 | 3.60 |
| S1/S5 composite - 1.50 g cm^-3^ | 53 | 15 | 25 | 7 | 3.53 |
| S2/S3/S4 composite - 1.50 g cm^-3^ | 54 | 13 | 25 | 8 | 4.15 |
| Literature data for powered MX-80^2^ | 51-59 | 7-16.5 | 28-32 | 4-5 | 3.1-7.4 |

^1^A ratio of NMR signal (%) of alkyl/*O*-alkyl carbon

**^2^** Data reported by: 1, 2, 4


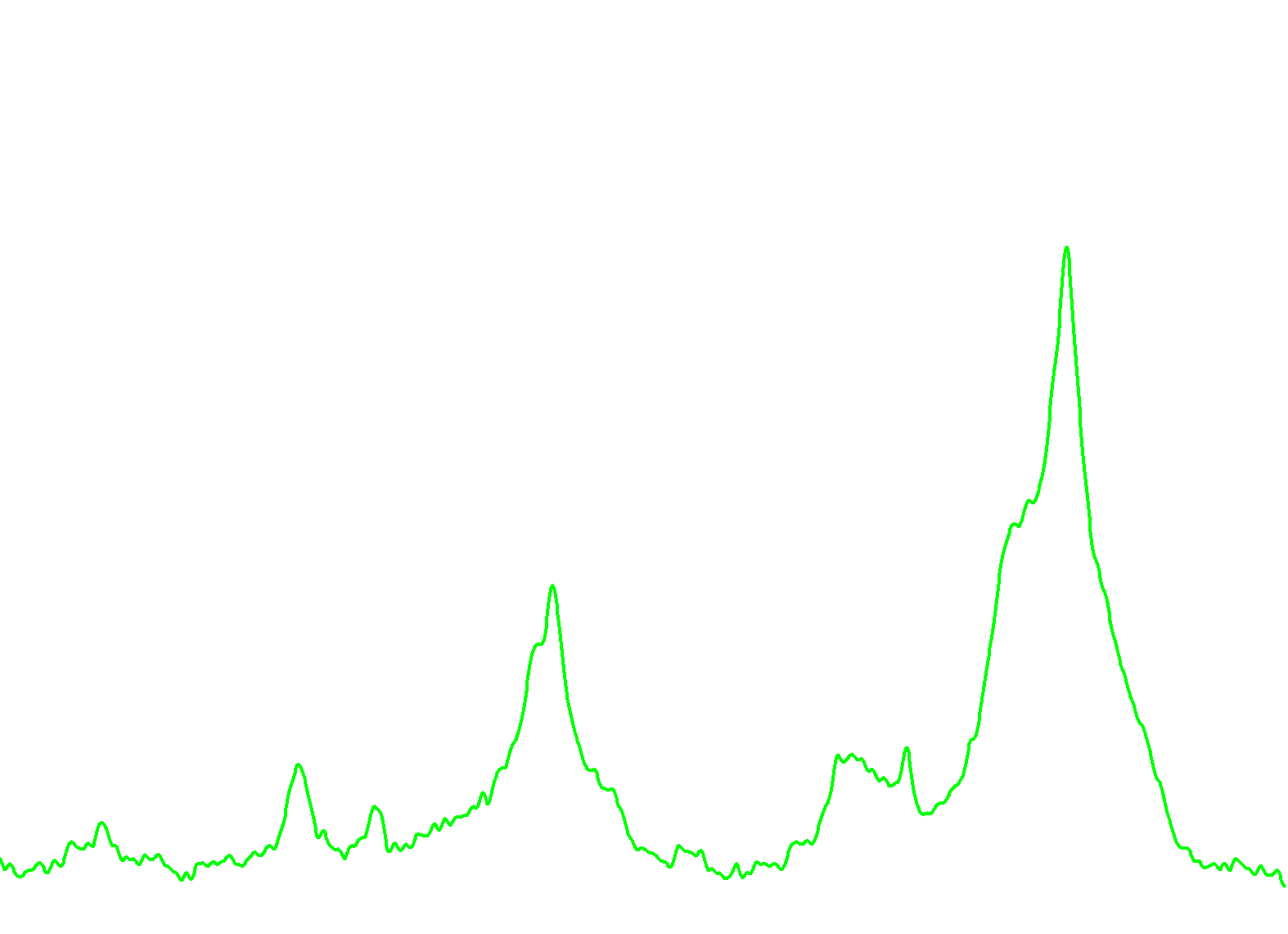

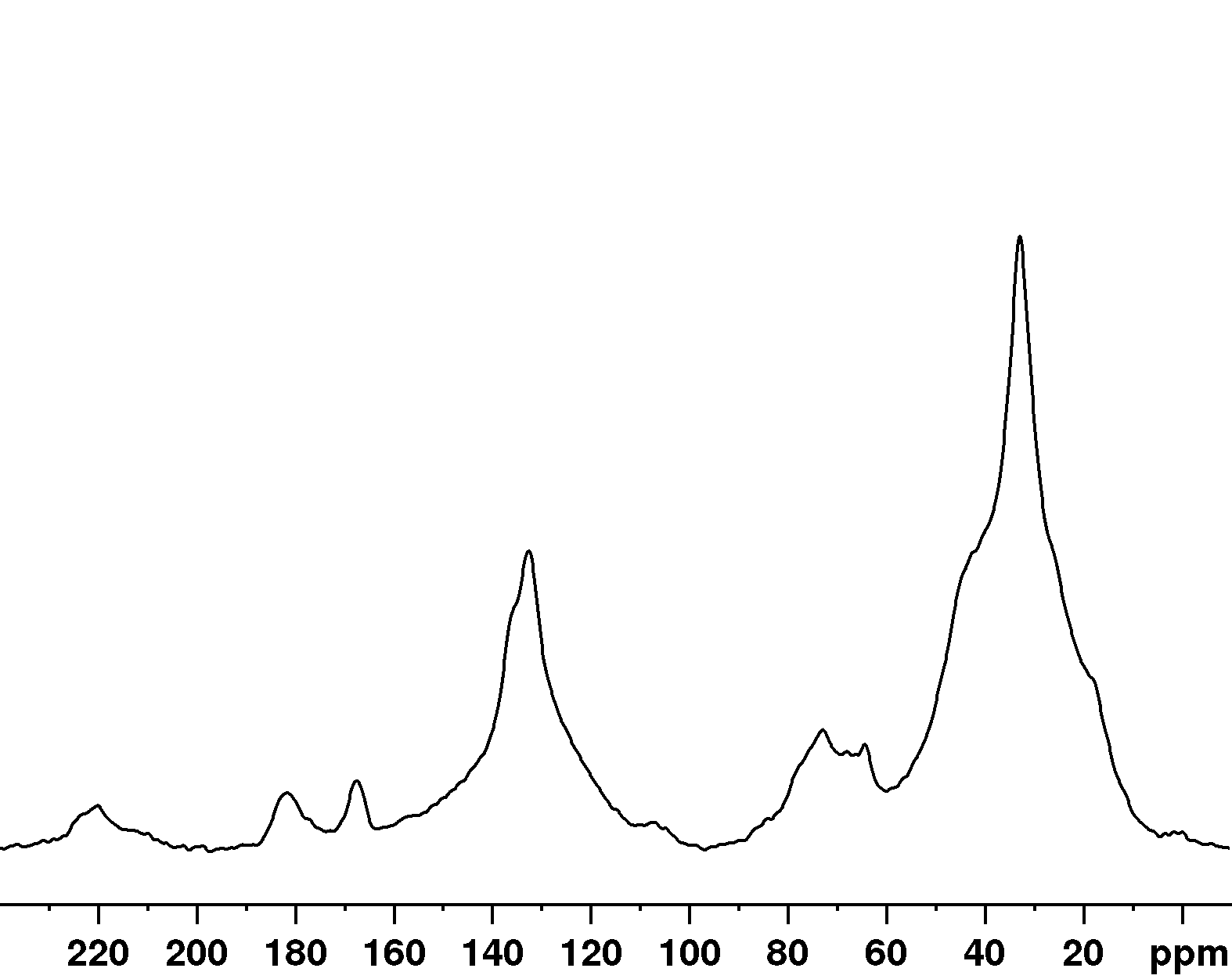

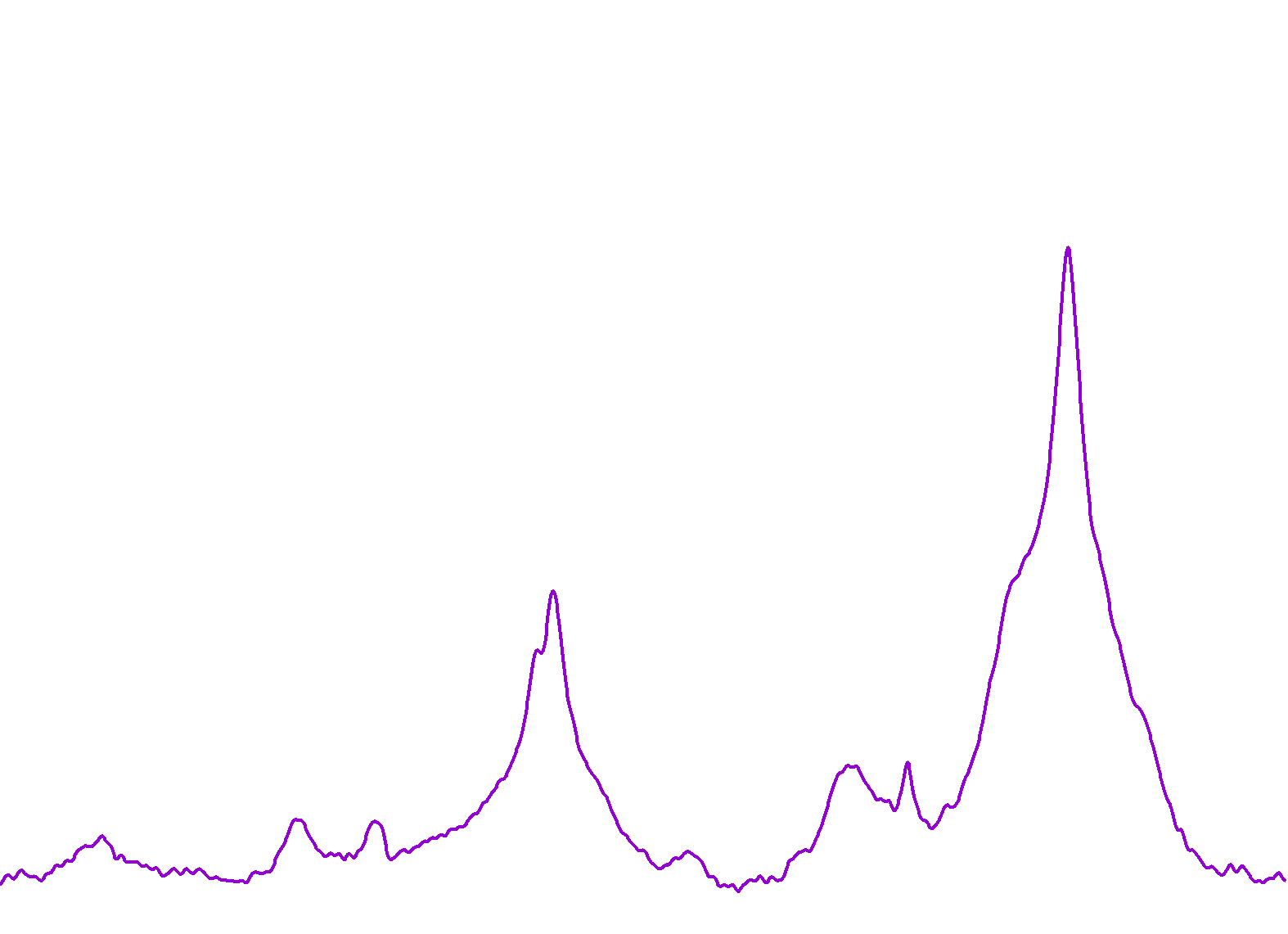

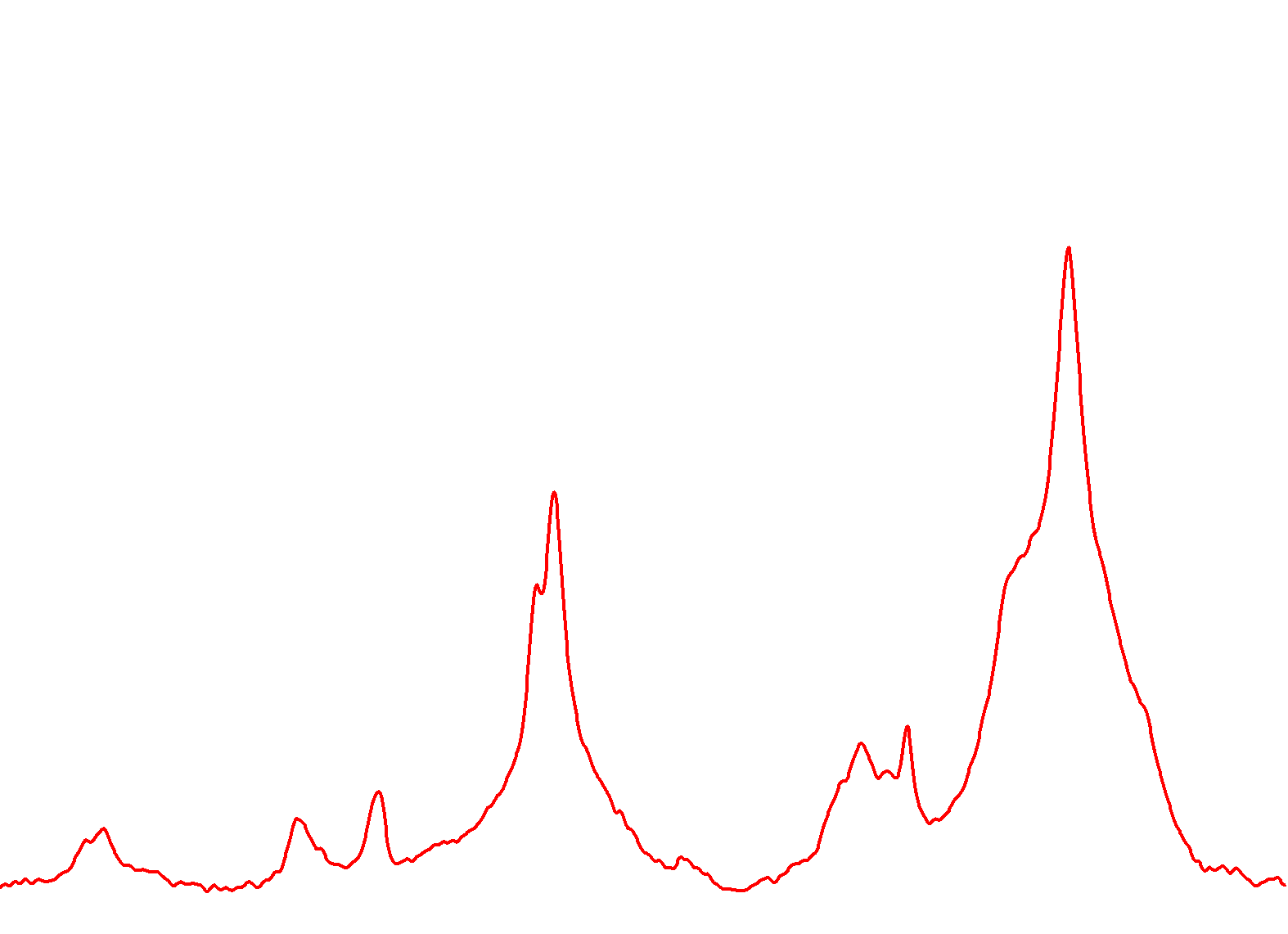

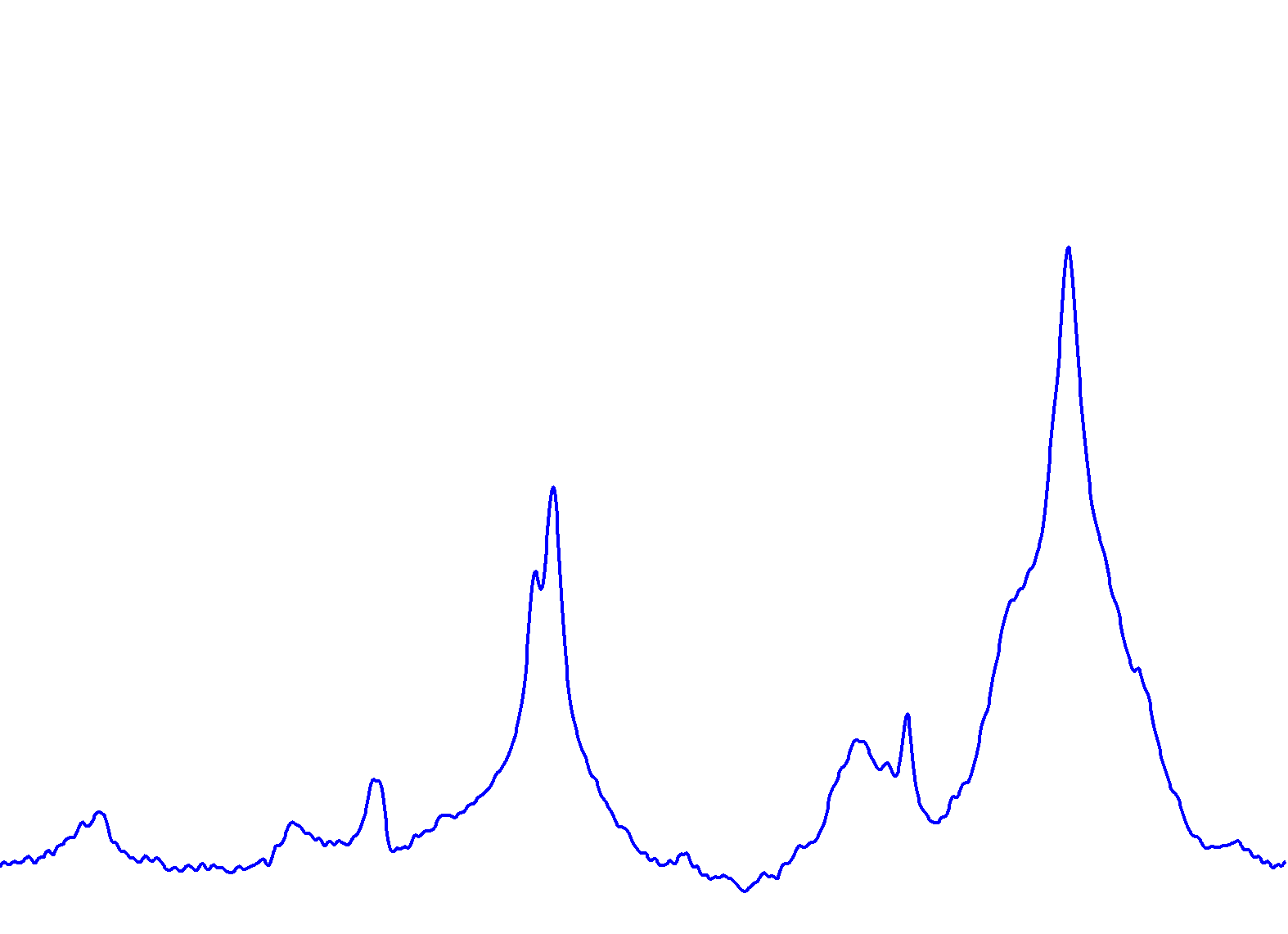


S2/S3/S4 composite – 1.50 g/cm^3^

S1/S5 composite – 1.50 g/cm^3^

S2/S3/S4 composite – 1.25 g/cm^3^

S1/S5 composite - 1.25 g/cm^3^

Reference (mix)

**^13^C Chemical Shift**

**Figure S3:** Solid-state ^13^C NMR spectra of MaCoTe module composite samples at two densities (1.25 and 1.50 g cm^-3^) in comparison to the reference material (mix of fine and coarse reference samples).

*Total Carbon, Inorganic Carbon, and Organic Carbon Analysis*

Carbon profiles of reference and composite bentonite samples are listed in Table S5. The values for both reference and composite bentonites are within the same range. However, other studies have reported higher total carbon values for powdered Wyoming type bentonites (MX-80) (1-4). The organic carbon (Table S5) is consistent with reports from other MX-80 bentonite clays however, the composite and reference bentonites are lower in inorganic carbon (1-4).

**Table S5:** Average (± standard deviation) carbon profiles of inner layer MaCoTe composites (S1 to S5; n=3) and starting materials (MX6 and MX7 composite; n=4).

| Sample Name | Total carbon (%) | Inorganic carbon (%) | Organic carbon (%) |
| --- | --- | --- | --- |
| MX6 and MX7 composite | 0.415 ± 0.013 | 0.183 ± 0.006 | 0.231 ± 0.017 |
| Inner layer composite – 1.25 g cm^-3^ | 0.355 ± 0.012 | 0.179 ± 0.017 | 0.176 ± 0.006 |
| Inner layer composite - 1.50 g cm^-3^ | 0.392 ± 0.012 | 0.187 ± 0.003 | 0.205 ± 0.012 |
| Literature data for powdered MX-80* | 0.555 - 0.98 | 0.437 - 0.745 | 0.095 – 0.235 |

* Data reported by: 1, 2, 4. The organic carbon in the MX6 and MX7 composite (starting material) is statistically the same as the inner layer composites - 1.50 g cm^-3^ but greater than the inner layer composites - 1.25 g cm^-3^.

**Section III. Water Activity and Moisture Content**

Water activity of bentonite samples was determined using the fast mode on the WP4 Potentiometer (Meter Group, USA) following the manufacturer’s instructions. The instrument was calibrated before each use with a solution of known water potential (0.5 M KCl). Water activity was calculated using the following equation:

$$water activity =exp\frac{water potential* molecular weight of water}{temperature* gas constant}$$

Subsequently, bentonite samples were heated at 110°C for 24 h to determine moisture content using the following equation:

$$moisture content (\%)=\left( \frac{wet weight-dry weight}{wet weight} \right)\times100$$

**Table S6.** Water activity and moisture content of bentonite samples from years 5 and 7. Standard deviation of 5 replicates is shown.

| Dry density (g cm^-3^) | Layer type | Moisture (%)  Year 5 | Water activity  Year 5 | Moisture (%)  Year 7 | Water activity  Year 7 |
| --- | --- | --- | --- | --- | --- |
| 1.25 | Outer layer | NA | NA | 46.47±2.98 | 0.990±0.001 |
| 1.25 | Inner layer | 44.45±2.28 | 0.983±0.006 | 39.84±0.61 | 0.987±0.001 |
| 1.50 | Outer layer | NA | NA | 34.63±0.85 | 0.975±0.006 |
| 1.50 | Inner layer | 31.67±1.77 | 0.954±0.005 | 30.31±1.31 | 0.960±0.008 |

**Section IV.** **Year 7 Bentonite clay nutrient content**

The nutrient content of S1 to S5 composites of both density samples from year 7 were analyzed by University of Guelph’s, Ontario, Agriculture and Food Laboratory. The results are summarized in table S7.

**Table S7.** Nutrient content of the starting materials and composite year 7 bentonite samples.

| Sample | **Ca**^1^ (mg/kg dry) | **NH_4_-N**^2^ (mg/kg dry) | **NO_3_-N**^2^ (mg/kg dry) | **Na**^1^ (mg/kg dry) | **P**^3^ (mg/kg dry) | **Mg**^1^ (mg/kg dry) | **K**^1^ (mg/kg dry) | pH | **Total N**^4^ (% dry) | **Total S**^5^ (% dry) |
| --- | --- | --- | --- | --- | --- | --- | --- | --- | --- | --- |
| Inner layer S1 to S5 composites, 1.50 g cm^-3^ | 6120 | 99.1 | 2.40 | 9274 | 8.8 | 1000 | 450 | 8.4 | 0.03 | 0.177 |
| Inner layer S1 to S5 composites, 1.25 g cm^-3^ | 5850 | 82.3 | 2.20 | 10452 | 7.4 | 1100 | 390 | 8.5 | 0.03 | 0.168 |
| MX6 | 6380 | 41.4 | 14.3 | 12479 | 9.2 | 560 | 470 | 8.5 | <0.02 | 0.24 |
| MX7 | 6050 | 42.7 | 13.2 | 12722 | 9.4 | 600 | 440 | 8.4 | <0.02 | 0.25 |

^1^ NH_4_^+^ extractable

^2^ KCl extractable

^3^ NaHCO_3_ extractable

^4^ Combustion

^5^ Total acid digestion

**Section V. Bentonite clay mineralogy**

Mineral compositions of the starting bentonite materials (MX6 and MX7) and composites of sections 1 to 5 of the two densities (1.25 and 1.50 g cm^-3^) of year 7 inner layer samples were assessed by Activation Laboratories ltd. *Hamilton, ON) via quantitative X-ray diffraction and clay speciation analyses. Mineral compositions of the MX6 and MX7 were similar and dominated by 2:1 phyllosilicate mineral montmorillonite (~75%), followed by plagioclase (~12%), Quartz (<5%), and K-feldspar (<5%) and remained similar in the inner layers after 7 years (Table S8).

**Table S8.** Mineral compositions (wt %) of bentonite samples. A mixture of MX6 and MX7 was used to prepare the modules before emplacement in the borehole. For the Year 7 modules, a composite sample of inner bentonite from sections 1 (top) to 5 (bottom) was prepared for each dry density.

| Mineral | MX6 | MX7 | Year 7 1.25 g cm^-3^ | Year 7 1.50 g cm^-3^ |
| --- | --- | --- | --- | --- |
| Montmorillonite | 77.6 | 75.2 | 77.0 | 77.3 |
| Illite-Smectite | n.d. | n.d. | n.d. | n.d. |
| Kaolinite | n.d. | n.d. | n.d. | n.d. |
| Quartz | 4.7 | 4.2 | 5.3 | 4.5 |
| Cristobalite | 1.7 | 1.1 | 2.1 | 1.9 |
| Plagioclase | 11.1 | 12.1 | 10.0 | 10.0 |
| K feldspar | 3.0 | 4.5 | 4.5 | 4.0 |
| Mica | 1.0 | 1.8 | n.d. | 1.4 |
| Chlorite | n.d. | n.d. | n.d. | n.d. |
| Clinoptilolite | n.d. | n.d. | n.d. | n.d. |
| Calcite | 0.4 | n.d. | 1.1 | 0.9 |
| Dolomite | n.d. | n.d. | n.d. | n.d. |
| Gypsum | 0.5 | 0.8 | n.d. | n.d. |
| Pyrite | n.d. | 0.3 | trace | n.d. |
| Goethite | n.d. | n.d. | n.d. | n.d. |

**Other tables and figures**

**Table S9.** Results of Dunn’s test with Benjamini-Hochberg *p* value adjustment for PLFA abundance comparisons between outer and inner layers of borehole samples from years 0 (starting material), 1, 5, and 7. Significant differences are indicated by *.

| Group1 | Group2 | Z | P.unadj | P.adj | Significance |
| --- | --- | --- | --- | --- | --- |
| Starting material | Year 1 inner layer bentonite 1.25 | 1.1835 | 0.2366 | 0.5316 |  |
| Starting material | Year 1 outer layer bentonite 1.25 | 1.9621 | 0.04975 | 0.215583 |  |
| Starting material | Year 5 inner layer bentonite 1.25 | 0.4719 | 0.637 | 0.818105 |  |
| Starting material | Year 5 outer layer bentonite 1.25 | 2.2698 | 0.02322 | 0.164651 |  |
| Starting material | Year 7 inner layer bentonite 1.25 | 1.2936 | 0.1958 | 0.5316 |  |
| Starting material | Year 7 outer layer bentonite 1.25 | 3.7659 | 0.000166 | 0.012948 | * |
| Starting material | Year 1 inner layer bentonite 1.50 | 0.8257 | 0.409 | 0.725045 |  |
| Starting material | Year 1 outer layer bentonite 1.50 | 1.1127 | 0.2658 | 0.5316 |  |
| Starting material | Year 5 inner layer bentonite 1.50 | 0.9664 | 0.3339 | 0.6201 |  |
| Starting material | Year 5 outer layer bentonite 1.50 | 2.2249 | 0.02609 | 0.169585 |  |
| Starting material | Year 7 inner layer bentonite 1.50 | 1.5377 | 0.1241 | 0.41925 |  |
| Starting material | Year 7 outer layer bentonite 1.50 | 3.3579 | 0.000785 | 0.030627 | * |
| Year 1 inner layer bentonite 1.25 | Year 1 outer layer bentonite 1.25 | 0.8664 | 0.3863 | 0.70073 |  |
| Year 1 inner layer bentonite 1.25 | Year 5 inner layer bentonite 1.25 | 0.4944 | 0.621 | 0.818105 |  |
| Year 1 inner layer bentonite 1.25 | Year 5 outer layer bentonite 1.25 | 1.3035 | 0.1924 | 0.5316 |  |
| Year 1 inner layer bentonite 1.25 | Year 7 inner layer bentonite 1.25 | 0.1101 | 0.9123 | 0.982695 |  |
| Year 1 inner layer bentonite 1.25 | Year 7 outer layer bentonite 1.25 | 2.5183 | 0.01179 | 0.15327 |  |
| Year 1 inner layer bentonite 1.25 | Year 1 inner layer bentonite 1.50 | 0.3578 | 0.7205 | 0.886763 |  |
| Year 1 inner layer bentonite 1.25 | Year 1 outer layer bentonite 1.50 | 0.01699 | 0.9864 | 1 |  |
| Year 1 inner layer bentonite 1.25 | Year 5 inner layer bentonite 1.50 | 0 | 1 | 1 |  |
| Year 1 inner layer bentonite 1.25 | Year 5 outer layer bentonite 1.50 | 1.2585 | 0.2082 | 0.5316 |  |
| Year 1 inner layer bentonite 1.25 | Year 7 inner layer bentonite 1.50 | 0.2901 | 0.7717 | 0.912009 |  |
| Year 1 inner layer bentonite 1.25 | Year 7 outer layer bentonite 1.50 | 2.1744 | 0.02967 | 0.17802 |  |
| Year 1 outer layer bentonite 1.25 | Year 5 inner layer bentonite 1.25 | 1.1939 | 0.2325 | 0.5316 |  |
| Year 1 outer layer bentonite 1.25 | Year 5 outer layer bentonite 1.25 | 0.5117 | 0.6089 | 0.818105 |  |
| Year 1 outer layer bentonite 1.25 | Year 7 inner layer bentonite 1.25 | 0.7645 | 0.4446 | 0.753887 |  |
| Year 1 outer layer bentonite 1.25 | Year 7 outer layer bentonite 1.25 | 1.4071 | 0.1594 | 0.497328 |  |
| Year 1 outer layer bentonite 1.25 | Year 1 inner layer bentonite 1.50 | 1.1977 | 0.231 | 0.5316 |  |
| Year 1 outer layer bentonite 1.25 | Year 1 outer layer bentonite 1.50 | 0.7946 | 0.4269 | 0.73996 |  |
| Year 1 outer layer bentonite 1.25 | Year 5 inner layer bentonite 1.50 | 0.7249 | 0.4685 | 0.754053 |  |
| Year 1 outer layer bentonite 1.25 | Year 5 outer layer bentonite 1.50 | 0.469 | 0.639 | 0.818105 |  |
| Year 1 outer layer bentonite 1.25 | Year 7 inner layer bentonite 1.50 | 0.6396 | 0.5224 | 0.7836 |  |
| Year 1 outer layer bentonite 1.25 | Year 7 outer layer bentonite 1.50 | 1.1467 | 0.2515 | 0.5316 |  |
| Year 5 inner layer bentonite 1.25 | Year 5 outer layer bentonite 1.25 | 1.557 | 0.1195 | 0.41925 |  |
| Year 5 inner layer bentonite 1.25 | Year 7 inner layer bentonite 1.25 | 0.5843 | 0.559 | 0.818105 |  |
| Year 5 inner layer bentonite 1.25 | Year 7 outer layer bentonite 1.25 | 2.5309 | 0.01138 | 0.15327 |  |
| Year 5 inner layer bentonite 1.25 | Year 1 inner layer bentonite 1.50 | 0.2023 | 0.8397 | 0.935666 |  |
| Year 5 inner layer bentonite 1.25 | Year 1 outer layer bentonite 1.50 | 0.4833 | 0.6289 | 0.818105 |  |
| Year 5 inner layer bentonite 1.25 | Year 5 inner layer bentonite 1.50 | 0.4282 | 0.6685 | 0.841016 |  |
| Year 5 inner layer bentonite 1.25 | Year 5 outer layer bentonite 1.50 | 1.5181 | 0.129 | 0.41925 |  |
| Year 5 inner layer bentonite 1.25 | Year 7 inner layer bentonite 1.50 | 0.7444 | 0.4566 | 0.754053 |  |
| Year 5 inner layer bentonite 1.25 | Year 7 outer layer bentonite 1.50 | 2.2698 | 0.02322 | 0.164651 |  |
| Year 5 outer layer bentonite 1.25 | Year 7 inner layer bentonite 1.25 | 1.2136 | 0.2249 | 0.5316 |  |
| Year 5 outer layer bentonite 1.25 | Year 7 outer layer bentonite 1.25 | 0.6699 | 0.5029 | 0.771741 |  |
| Year 5 outer layer bentonite 1.25 | Year 1 inner layer bentonite 1.50 | 1.5956 | 0.1106 | 0.41925 |  |
| Year 5 outer layer bentonite 1.25 | Year 1 outer layer bentonite 1.50 | 1.2224 | 0.2216 | 0.5316 |  |
| Year 5 outer layer bentonite 1.25 | Year 5 inner layer bentonite 1.50 | 1.1288 | 0.259 | 0.5316 |  |
| Year 5 outer layer bentonite 1.25 | Year 5 outer layer bentonite 1.50 | 0.03892 | 0.969 | 1 |  |
| Year 5 outer layer bentonite 1.25 | Year 7 inner layer bentonite 1.50 | 1.1166 | 0.2642 | 0.5316 |  |
| Year 5 outer layer bentonite 1.25 | Year 7 outer layer bentonite 1.50 | 0.4719 | 0.637 | 0.818105 |  |
| Year 7 inner layer bentonite 1.25 | Year 7 outer layer bentonite 1.25 | 2.4023 | 0.01629 | 0.164651 |  |
| Year 7 inner layer bentonite 1.25 | Year 1 inner layer bentonite 1.50 | 0.4679 | 0.6398 | 0.818105 |  |
| Year 7 inner layer bentonite 1.25 | Year 1 outer layer bentonite 1.50 | 0.08494 | 0.9323 | 0.982695 |  |
| Year 7 inner layer bentonite 1.25 | Year 5 inner layer bentonite 1.50 | 0.08989 | 0.9284 | 0.982695 |  |
| Year 7 inner layer bentonite 1.25 | Year 5 outer layer bentonite 1.50 | 1.1686 | 0.2426 | 0.5316 |  |
| Year 7 inner layer bentonite 1.25 | Year 7 inner layer bentonite 1.50 | 0.1741 | 0.8618 | 0.946766 |  |
| Year 7 inner layer bentonite 1.25 | Year 7 outer layer bentonite 1.50 | 2.0643 | 0.03899 | 0.210692 |  |
| Year 7 outer layer bentonite 1.25 | Year 1 inner layer bentonite 1.50 | 2.8955 | 0.003786 | 0.098436 |  |
| Year 7 outer layer bentonite 1.25 | Year 1 outer layer bentonite 1.50 | 2.2955 | 0.02171 | 0.164651 |  |
| Year 7 outer layer bentonite 1.25 | Year 5 inner layer bentonite 1.50 | 2.0192 | 0.04347 | 0.210692 |  |
| Year 7 outer layer bentonite 1.25 | Year 5 outer layer bentonite 1.50 | 0.7165 | 0.4737 | 0.754053 |  |
| Year 7 outer layer bentonite 1.25 | Year 7 inner layer bentonite 1.50 | 2.3634 | 0.01811 | 0.164651 |  |
| Year 7 outer layer bentonite 1.25 | Year 7 outer layer bentonite 1.50 | 0.2263 | 0.821 | 0.928087 |  |
| Year 1 inner layer bentonite 1.50 | Year 1 outer layer bentonite 1.50 | 0.3483 | 0.7276 | 0.886763 |  |
| Year 1 inner layer bentonite 1.50 | Year 5 inner layer bentonite 1.50 | 0.2922 | 0.7702 | 0.912009 |  |
| Year 1 inner layer bentonite 1.50 | Year 5 outer layer bentonite 1.50 | 1.5507 | 0.121 | 0.41925 |  |
| Year 1 inner layer bentonite 1.50 | Year 7 inner layer bentonite 1.50 | 0.6673 | 0.5046 | 0.771741 |  |
| Year 1 inner layer bentonite 1.50 | Year 7 outer layer bentonite 1.50 | 2.5322 | 0.01133 | 0.15327 |  |
| Year 1 outer layer bentonite 1.50 | Year 5 inner layer bentonite 1.50 | 0.01421 | 0.9887 | 1 |  |
| Year 1 outer layer bentonite 1.50 | Year 5 outer layer bentonite 1.50 | 1.1797 | 0.2381 | 0.5316 |  |
| Year 1 outer layer bentonite 1.50 | Year 7 inner layer bentonite 1.50 | 0.2487 | 0.8036 | 0.928087 |  |
| Year 1 outer layer bentonite 1.50 | Year 7 outer layer bentonite 1.50 | 1.9961 | 0.04592 | 0.210692 |  |
| Year 5 inner layer bentonite 1.50 | Year 5 outer layer bentonite 1.50 | 1.0899 | 0.2758 | 0.53781 |  |
| Year 5 inner layer bentonite 1.50 | Year 7 inner layer bentonite 1.50 | 0.2326 | 0.8161 | 0.928087 |  |
| Year 5 inner layer bentonite 1.50 | Year 7 outer layer bentonite 1.50 | 1.7754 | 0.07583 | 0.311302 |  |
| Year 5 outer layer bentonite 1.50 | Year 7 inner layer bentonite 1.50 | 1.0701 | 0.2846 | 0.541434 |  |
| Year 5 outer layer bentonite 1.50 | Year 7 outer layer bentonite 1.50 | 0.5169 | 0.6052 | 0.818105 |  |
| Year 7 inner layer bentonite 1.50 | Year 7 outer layer bentonite 1.50 | 2.0019 | 0.0453 | 0.210692 |  |

**Table S10.** Results of pairwise PERMANOVA using the *qiime diversity beta-group-significance* function and Bray-Curtis distance matrix.

| Group 1 | Group 2 | Sample size | Permutations | pseudo-F | *p*-value |
| --- | --- | --- | --- | --- | --- |
| Starting material | Bentonite inner layer | 43 | 999 | 2.020415 | 0.001 |
| Starting material | Bentonite outer layer | 41 | 999 | 9.028171 | 0.001 |
| Starting material | Transport flask fluid | 23 | 999 | 7.026724 | 0.001 |
| Starting material | Borehole fluid | 18 | 999 | 5.061773 | 0.001 |
| Bentonite inner layer | Bentonite outer layer | 60 | 999 | 16.25945 | 0.001 |
| Bentonite inner layer | Transport flask fluid | 42 | 999 | 13.80546 | 0.001 |
| Bentonite inner layer | Borehole fluid | 37 | 999 | 9.657342 | 0.001 |
| Bentonite outer layer | Transport flask fluid | 40 | 999 | 16.35358 | 0.001 |
| Bentonite outer layer | Borehole fluid | 35 | 999 | 12.35486 | 0.001 |
| Transport flask fluid | Borehole fluid | 17 | 999 | 4.434218 | 0.001 |

**Table S11.** Results of Dunn’s test with Benjamini-Hochberg *p* value adjustment for quantitative PCR abundance estimates in outer and inner layers of borehole module bentonite samples from years 5 (Y5) and 7 (Y7) in comparison to starting material extracted along with Y5 or Y7 samples.

| Group1 | Group2 | Z | P.unadj | P.adj | Significance |
| --- | --- | --- | --- | --- | --- |
| Y5 inner layer bentonite 1.25 | Y5 inner layer bentonite 1.50 | 0.714 | 0.476 | 0.636 |  |
| Y5 inner layer bentonite 1.25 | Y5 outer layer bentonite 1.25 | -1.285 | 0.199 | 0.355 |  |
| Y5 inner layer bentonite 1.50 | Y5 outer layer bentonite 1.25 | -1.818 | 0.069 | 0.185 |  |
| Y5 inner layer bentonite 1.25 | Y5 outer layer bentonite 1.50 | 0.546 | 0.585 | 0.740 |  |
| Y5 inner layer bentonite 1.50 | Y5 outer layer bentonite 1.50 | -0.151 | 0.880 | 0.910 |  |
| Y5 outer layer bentonite 1.25 | Y5 outer layer bentonite 1.50 | 1.660 | 0.097 | 0.226 |  |
| Y5 inner layer bentonite 1.25 | Y5 starting material | 0.500 | 0.617 | 0.739 |  |
| Y5 inner layer bentonite 1.50 | Y5 starting material | -0.226 | 0.821 | 0.879 |  |
| Y5 outer layer bentonite 1.25 | Y5 starting material | 1.669 | 0.095 | 0.234 |  |
| Y5 outer layer bentonite 1.50 | Y5 starting material | -0.068 | 0.945 | 0.956 |  |
| Y7 inner layer bentonite 1.25 | Y7 inner layer bentonite 1.50 | 0.831 | 0.406 | 0.606 |  |
| Y7 inner layer bentonite 1.25 | Y7 outer layer bentonite 1.25 | 0.015 | 0.988 | 0.988 |  |
| Y7 inner layer bentonite 1.50 | Y7 outer layer bentonite 1.25 | -0.816 | 0.414 | 0.608 |  |
| Y7 inner layer bentonite 1.25 | Y7 outer layer bentonite 1.50 | -0.257 | 0.797 | 0.864 |  |
| Y7 inner layer bentonite 1.50 | Y7 outer layer bentonite 1.50 | -1.088 | 0.277 | 0.458 |  |
| Y7 outer layer bentonite 1.25 | Y7 outer layer bentonite 1.50 | -0.272 | 0.786 | 0.872 |  |
| Y7 inner layer bentonite 1.25 | Y7 starting material | 1.393 | 0.164 | 0.331 |  |
| Y7 inner layer bentonite 1.50 | Y7 starting material | 0.525 | 0.600 | 0.738 |  |
| Y7 outer layer bentonite 1.25 | Y7 starting material | 1.377 | 0.168 | 0.333 |  |
| Y7 outer layer bentonite 1.50 | Y7 starting material | 1.661 | 0.097 | 0.232 |  |

**Table S12.** Results of Dunn’s test with Benjamini-Hochberg *p* value adjustment for PLFA based microbial abundance estimates in outer and inner layers of borehole module bentonite samples from years 5 (Y5) and 7 (Y7) in comparison to starting material extracted along with Y5 or Y7 samples.

| Group1 | Group2 | Z | P.unadj | P.adj | Significance |
| --- | --- | --- | --- | --- | --- |
| Y5 starting material | Y5 inner layer bentonite 1.25 | -0.545 | 0.58579 | 0.8329179 |  |
| Y5 starting material | Y5 inner layer bentonite 1.50 | -0.973 | 0.33049 | 0.62655744 |  |
| Y5 inner layer bentonite 1.25 | Y5 inner layer bentonite 1.50 | -0.428 | 0.66852 | 0.83336571 |  |
| Y5 starting material | Y5 outer layer bentonite 1.25 | -2.102 | 0.03556 | 0.23112639 |  |
| Y5 inner layer bentonite 1.25 | Y5 outer layer bentonite 1.25 | -1.557 | 0.11947 | 0.43487439 |  |
| Y5 inner layer bentonite 1.50 | Y5 outer layer bentonite 1.25 | -1.129 | 0.25897 | 0.5747922 |  |
| Y5 starting material | Y5 outer layer bentonite 1.50 | -2.063 | 0.03911 | 0.22244108 |  |
| Y5 inner layer bentonite 1.25 | Y5 outer layer bentonite 1.50 | -1.518 | 0.129 | 0.43476434 |  |
| Y5 inner layer bentonite 1.50 | Y5 outer layer bentonite 1.50 | -1.09 | 0.27576 | 0.57031737 |  |
| Y5 outer layer bentonite 1.25 | Y5 outer layer bentonite 1.50 | 0.0389 | 0.96895 | 1 |  |
| Y7 starting material | Y7 inner layer bentonite 1.25 | -0.899 | 0.36869 | 0.68470679 |  |
| Y7 starting material | Y7 inner layer bentonite 1.50 | -1.07 | 0.28459 | 0.55102189 |  |
| Y7 inner layer bentonite 1.25 | Y7 inner layer bentonite 1.50 | -0.174 | 0.8618 | 0.93362147 |  |
| Y7 starting material | Y7 outer layer bentonite 1.25 | -2.857 | 0.00428 | 0.09742079 |  |
| Y7 inner layer bentonite 1.25 | Y7 outer layer bentonite 1.25 | -2.402 | 0.01629 | 0.16474644 |  |
| Y7 inner layer bentonite 1.50 | Y7 outer layer bentonite 1.25 | -2.363 | 0.01811 | 0.16480312 |  |
| Y7 starting material | Y7 outer layer bentonite 1.50 | -2.584 | 0.00975 | 0.17752154 |  |
| Y7 inner layer bentonite 1.25 | Y7 outer layer bentonite 1.50 | -2.064 | 0.03899 | 0.23653091 |  |
| Y7 inner layer bentonite 1.50 | Y7 outer layer bentonite 1.50 | -2.002 | 0.0453 | 0.22899764 |  |
| Y7 outer layer bentonite 1.25 | Y7 outer layer bentonite 1.50 | 0.2263 | 0.82097 | 0.91107353 |  |

**Table S13.** Results of Dunn’s test with Benjamini-Hochberg *p* value adjustment for aerobic heterotroph abundance estimates in outer and inner layers of borehole module bentonite samples from years 5 (Y5) and 7 (Y7) in comparison to starting material extracted along with Y5 or Y7 samples. Significant differences below α of 0.01 are indicated by ** and between 0.01 and 0.05 are indicated by *.

| Group1 | Group2 | Z | P.unadj | P.adj | Significance |
| --- | --- | --- | --- | --- | --- |
| Y5 inner layer bentonite 1.25 | Y5 inner layer bentonite 1.50 | 0.629 | 0.530 | 0.662 |  |
| Y5 inner layer bentonite 1.25 | Y5 outer layer bentonite 1.25 | -2.938 | 0.003 | 0.017 | * |
| Y5 inner layer bentonite 1.25 | Y5 outer layer bentonite 1.50 | 0.545 | 0.586 | 0.715 |  |
| Y5 inner layer bentonite 1.25 | Y5 starting material | -0.906 | 0.365 | 0.532 |  |
| Y5 inner layer bentonite 1.50 | Y5 outer layer bentonite 1.25 | -3.531 | 0.000 | 0.005 | ** |
| Y5 inner layer bentonite 1.50 | Y5 outer layer bentonite 1.50 | -0.084 | 0.933 | 0.951 |  |
| Y5 inner layer bentonite 1.50 | Y5 starting material | -1.585 | 0.113 | 0.242 |  |
| Y5 outer layer bentonite 1.25 | Y5 outer layer bentonite 1.50 | 3.452 | 0.001 | 0.005 | ** |
| Y5 outer layer bentonite 1.25 | Y5 starting material | 2.298 | 0.022 | 0.069 |  |
| Y5 outer layer bentonite 1.50 | Y5 starting material | -1.495 | 0.135 | 0.278 |  |
| Y7 inner layer bentonite 1.25 | Y7 inner layer bentonite 1.50 | 1.661 | 0.097 | 0.211 |  |
| Y7 inner layer bentonite 1.25 | Y7 outer layer bentonite 1.25 | -3.313 | 0.001 | 0.007 | ** |
| Y7 inner layer bentonite 1.25 | Y7 outer layer bentonite 1.50 | 0.440 | 0.660 | 0.770 |  |
| Y7 inner layer bentonite 1.25 | Y7 starting material | -1.274 | 0.203 | 0.367 |  |
| Y7 inner layer bentonite 1.50 | Y7 outer layer bentonite 1.25 | -4.975 | 0.000 | 0.000 | ** |
| Y7 inner layer bentonite 1.50 | Y7 outer layer bentonite 1.50 | -1.221 | 0.222 | 0.382 |  |
| Y7 inner layer bentonite 1.50 | Y7 starting material | -2.879 | 0.004 | 0.019 | * |
| Y7 outer layer bentonite 1.25 | Y7 outer layer bentonite 1.50 | 3.753 | 0.000 | 0.003 | ** |
| Y7 outer layer bentonite 1.25 | Y7 starting material | 1.927 | 0.054 | 0.135 |  |
| Y7 outer layer bentonite 1.50 | Y7 starting material | -1.699 | 0.089 | 0.200 |  |

**Table S14.** Results of Dunn’s test with Benjamini-Hochberg *p* value adjustment for anaerobic heterotroph abundance estimates in outer and inner layers of borehole module bentonite samples from years 5 (Y5) and 7 (Y7) in comparison to starting material extracted along with Y5 or Y7 samples. Significant differences below are indicated by *.

| Group1 | Group2 | Z | P.unadj | P.adj | Significance |
| --- | --- | --- | --- | --- | --- |
| Y5 inner layer bentonite 1.25 | Y5 inner layer bentonite 1.50 | -0.664 | 0.507 | 0.794 |  |
| Y5 inner layer bentonite 1.25 | Y5 outer layer bentonite 1.25 | 0.367 | 0.713 | 0.892 |  |
| Y5 inner layer bentonite 1.25 | Y5 outer layer bentonite 1.50 | 0.367 | 0.713 | 0.902 |  |
| Y5 inner layer bentonite 1.25 | Y5 starting material | -2.337 | 0.019 | 0.070 |  |
| Y5 inner layer bentonite 1.50 | Y5 outer layer bentonite 1.25 | 1.032 | 0.302 | 0.557 |  |
| Y5 inner layer bentonite 1.50 | Y5 outer layer bentonite 1.50 | 1.032 | 0.302 | 0.567 |  |
| Y5 inner layer bentonite 1.50 | Y5 starting material | -1.620 | 0.105 | 0.246 |  |
| Y5 outer layer bentonite 1.25 | Y5 outer layer bentonite 1.50 | 0.000 | 1.000 | 1.000 |  |
| Y5 outer layer bentonite 1.25 | Y5 starting material | -2.734 | 0.006 | 0.037 | * |
| Y5 outer layer bentonite 1.50 | Y5 starting material | -2.734 | 0.006 | 0.039 | * |
| Y7 inner layer bentonite 1.25 | Y7 inner layer bentonite 1.50 | 0.459 | 0.646 | 0.881 |  |
| Y7 inner layer bentonite 1.25 | Y7 outer layer bentonite 1.25 | 0.652 | 0.514 | 0.782 |  |
| Y7 inner layer bentonite 1.25 | Y7 outer layer bentonite 1.50 | 0.214 | 0.830 | 0.928 |  |
| Y7 inner layer bentonite 1.25 | Y7 starting material | -0.132 | 0.895 | 0.940 |  |
| Y7 inner layer bentonite 1.50 | Y7 outer layer bentonite 1.25 | 0.194 | 0.846 | 0.936 |  |
| Y7 inner layer bentonite 1.50 | Y7 outer layer bentonite 1.50 | -0.245 | 0.807 | 0.911 |  |
| Y7 inner layer bentonite 1.50 | Y7 starting material | -0.557 | 0.578 | 0.820 |  |
| Y7 outer layer bentonite 1.25 | Y7 outer layer bentonite 1.50 | -0.438 | 0.661 | 0.879 |  |
| Y7 outer layer bentonite 1.25 | Y7 starting material | -0.736 | 0.462 | 0.746 |  |
| Y7 outer layer bentonite 1.50 | Y7 starting material | -0.330 | 0.741 | 0.874 |  |

**Table S15.** Results of Dunn’s test with Benjamini-Hochberg *p* value adjustment for sulfate-reducing bacteria abundance estimates in outer and inner layers of borehole module bentonite samples from years 5 (Y5) and 7 (Y7) in comparison to starting material extracted along with Y5 or Y7 samples.

| Group1 | Group2 | Z | P.unadj | P.adj | Significance |
| --- | --- | --- | --- | --- | --- |
| Y5 inner layer bentonite 1.25 | Y5 inner layer bentonite 1.50 | -0.936 | 0.349 | 0.547 |  |
| Y5 inner layer bentonite 1.25 | Y5 outer layer bentonite 1.25 | -2.411 | 0.016 | 0.084 |  |
| Y5 inner layer bentonite 1.25 | Y5 outer layer bentonite 1.50 | -1.842 | 0.065 | 0.196 |  |
| Y5 inner layer bentonite 1.25 | Y5 starting material | -1.939 | 0.053 | 0.178 |  |
| Y5 inner layer bentonite 1.50 | Y5 outer layer bentonite 1.25 | -1.475 | 0.140 | 0.289 |  |
| Y5 inner layer bentonite 1.50 | Y5 outer layer bentonite 1.50 | -0.906 | 0.365 | 0.555 |  |
| Y5 inner layer bentonite 1.50 | Y5 starting material | -0.928 | 0.354 | 0.546 |  |
| Y5 outer layer bentonite 1.25 | Y5 outer layer bentonite 1.50 | 0.569 | 0.570 | 0.712 |  |
| Y5 outer layer bentonite 1.25 | Y5 starting material | 0.665 | 0.506 | 0.672 |  |
| Y5 outer layer bentonite 1.50 | Y5 starting material | 0.051 | 0.959 | 0.968 |  |
| Y7 inner layer bentonite 1.25 | Y7 inner layer bentonite 1.50 | -1.742 | 0.082 | 0.204 |  |
| Y7 inner layer bentonite 1.25 | Y7 outer layer bentonite 1.25 | -4.421 | 0.000 | 0.001 | ** |
| Y7 inner layer bentonite 1.25 | Y7 outer layer bentonite 1.50 | -3.896 | 0.000 | 0.003 | ** |
| Y7 inner layer bentonite 1.25 | Y7 starting material | -2.346 | 0.019 | 0.095 |  |
| Y7 inner layer bentonite 1.50 | Y7 outer layer bentonite 1.25 | -2.679 | 0.007 | 0.060 |  |
| Y7 inner layer bentonite 1.50 | Y7 outer layer bentonite 1.50 | -2.154 | 0.031 | 0.126 |  |
| Y7 inner layer bentonite 1.50 | Y7 starting material | -0.663 | 0.507 | 0.658 |  |
| Y7 outer layer bentonite 1.25 | Y7 outer layer bentonite 1.50 | 0.524 | 0.600 | 0.741 |  |
| Y7 outer layer bentonite 1.25 | Y7 starting material | 1.925 | 0.054 | 0.173 |  |
| Y7 outer layer bentonite 1.50 | Y7 starting material | 1.418 | 0.156 | 0.315 |  |


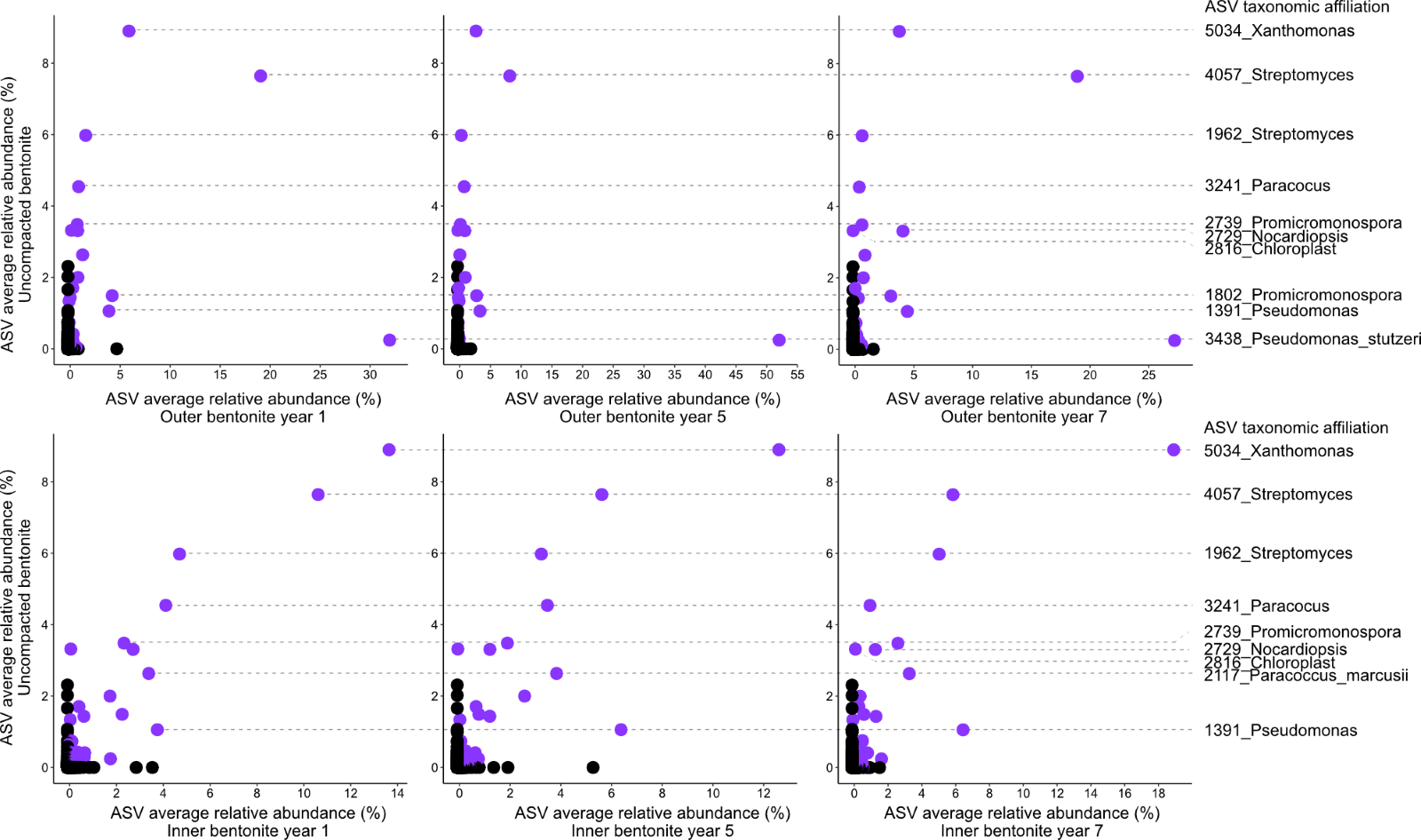


**Figure S4**. Scatter plot of ASVs detected in outer (top) or inner (bottom) bentonite samples after 1, 5, and 7 years of emplacement compared to the starting material. ASVs which were present in both, the starting material and the borehole module bentonite are highlighted in purple and black if absent.


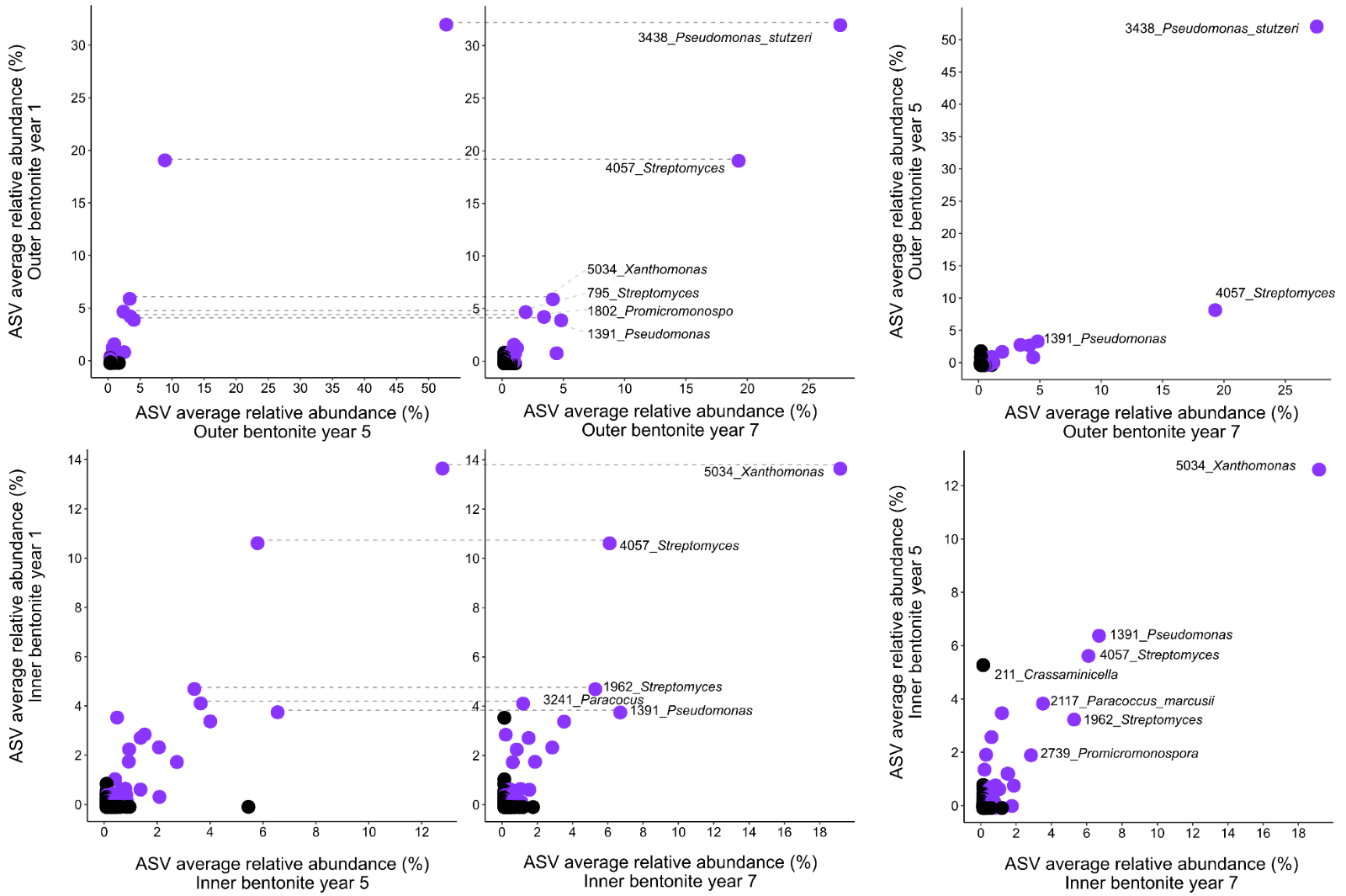


**Figure S5**. Scatter plot of ASVs detected in outer (top) or inner (bottom) bentonite samples after 1, 5, and 7 years of emplacement. ASVs which were present in both years are highlighted in purple and black if absent.


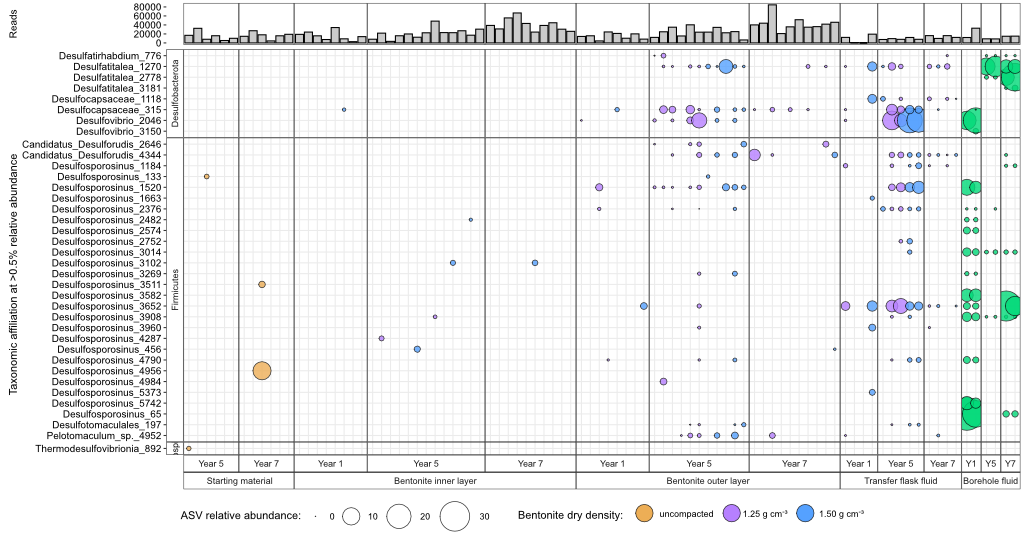


**Figure S6.** Bubble plot showing filtered 16S rRNA gene profiles of the borehole (BH) fluid, transfer flask (TF) fluid, outer and inner bentonite and starting material (MX6 and MX7; analyzed alongside year 5 and year 7 samples) for years 1 to 7. Only ASVs at or above 0.5% abundance are shown. To highlight putative SRBs, only ASVs affiliated with *Desulfosarcinaceae, Desulfocapsaceae, Desulfovibrionaceae, Desulforudaceae, Desulfitobacteriaceae, Desulfotomaculales, Desulfallas-Sporotomaculum*, and *Thermodesulfovibrionia* are shown.


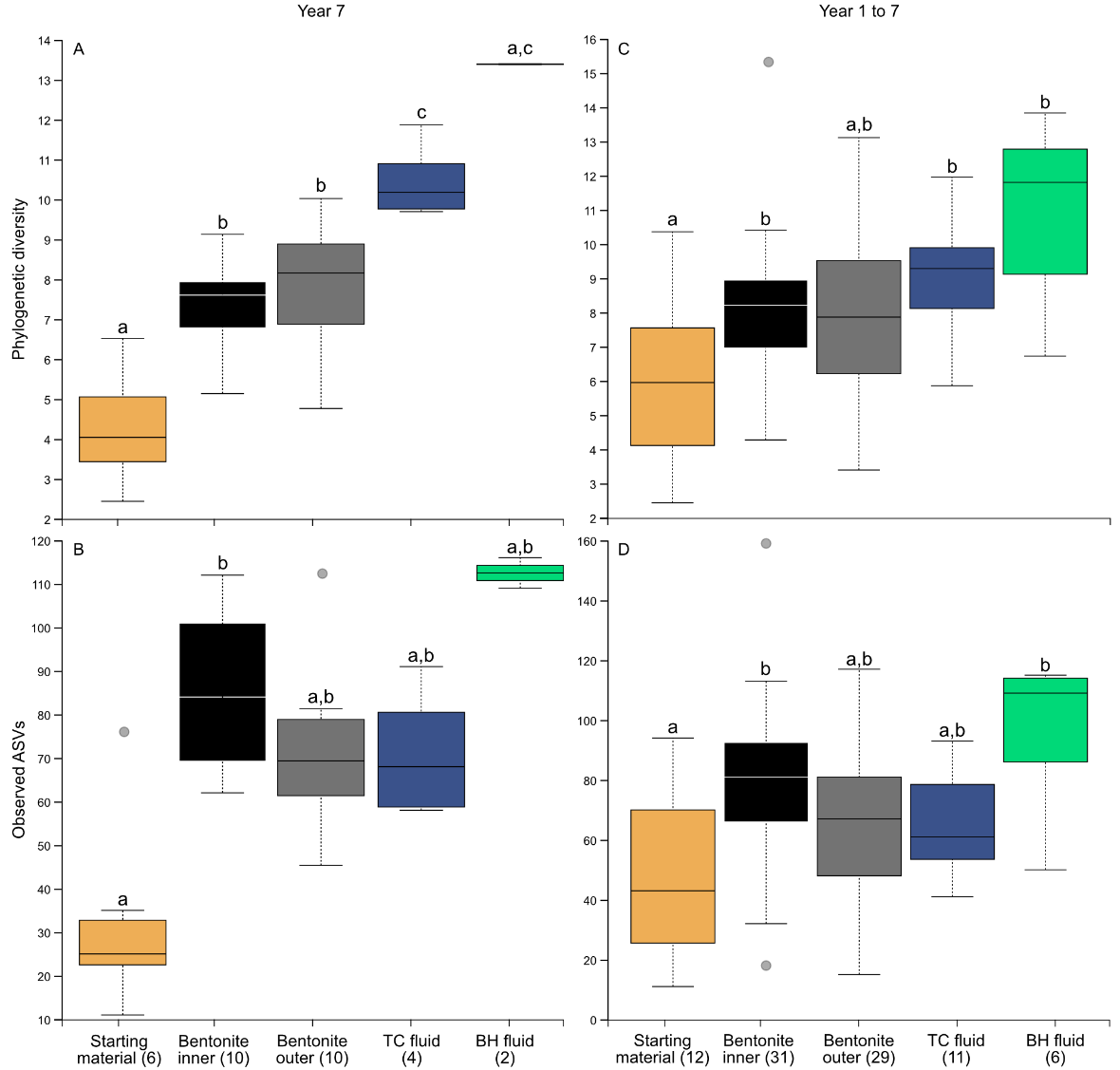


**Figure S7.** Faith’s phylogenetic diversity (top) and phylotype richness (bottom) within each sample type for Year 7 (left) or combined Year 1 to Year 7 (right) data. Metrics were calculated using data rarefied to 3,000 sequences per sample. The number of replicates per sample type is indicated in brackets. Different letters above box plots indicate significant differences (pairwise Kruskal-Wallis test with Benjamini-Hochberg *p* value adjustment, *p* < 0.05).


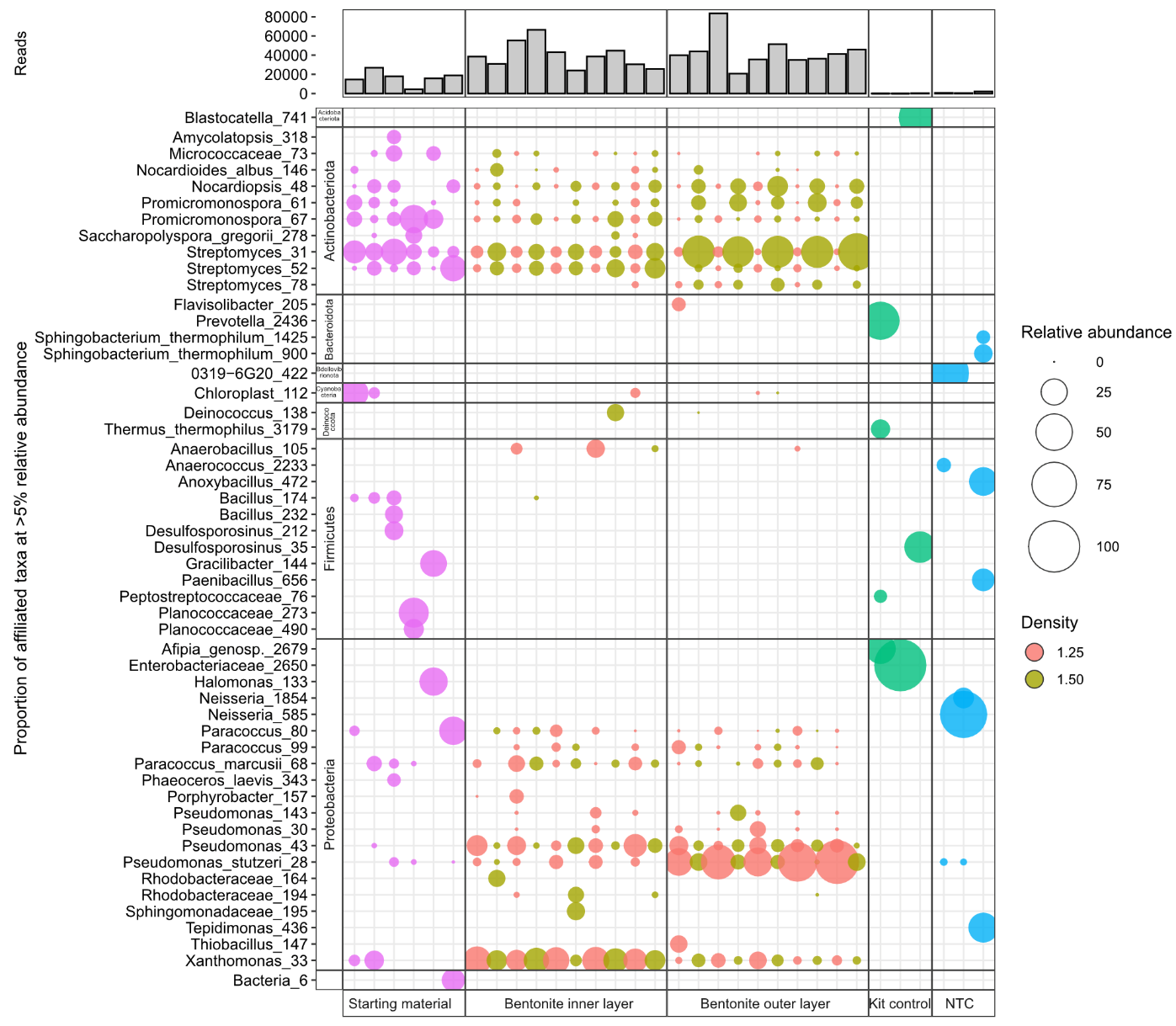


**Figure S8.** Bubble plot showing the DNeasy PowerMax Soil Kit controls and no-template controls (NTC) along Year 7 samples. Only ASVs at or above 5% relative abundance are shown.


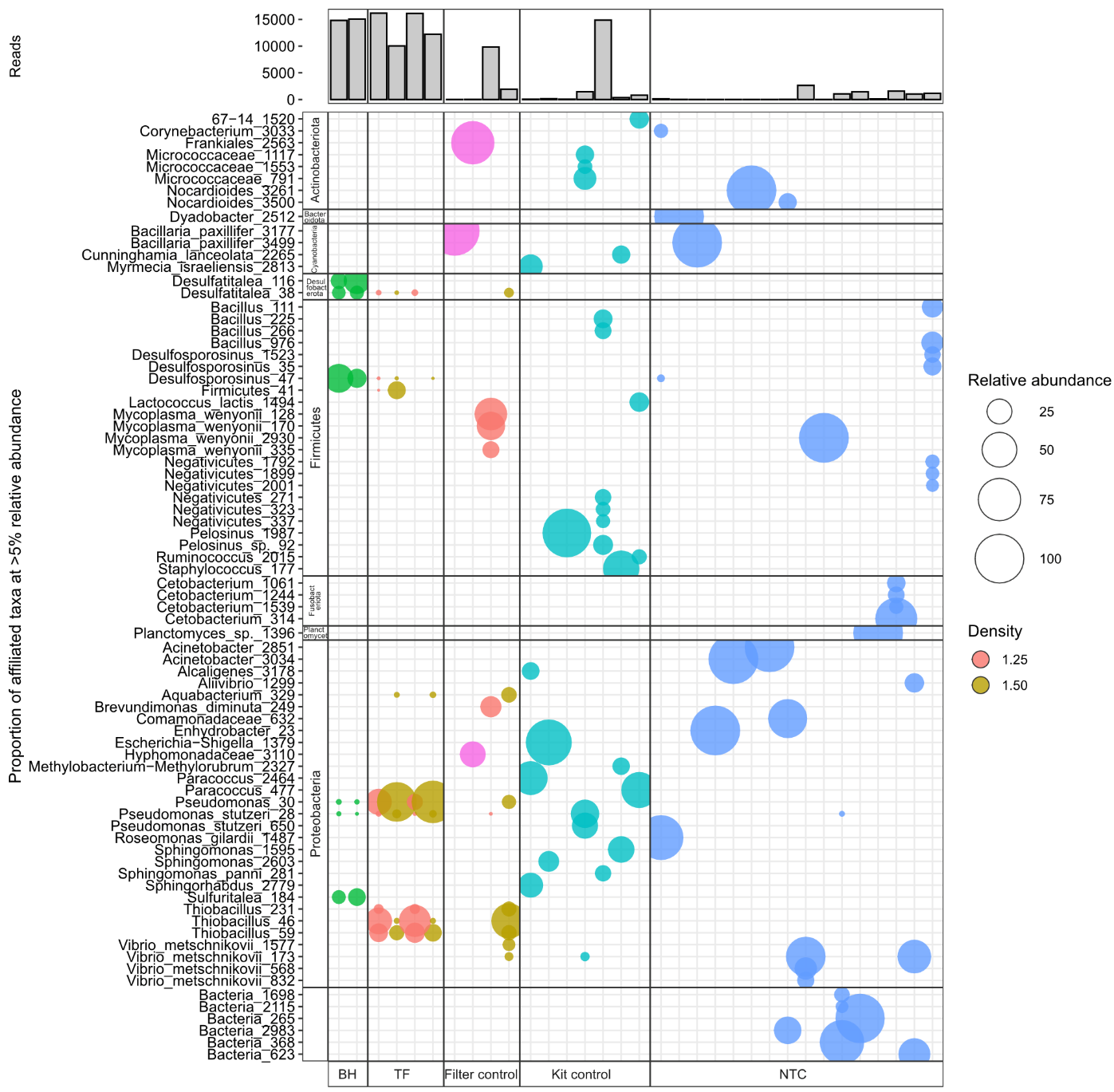


**Figure S9.** Bubble plot showing the DNeasy PowerSoil Pro Kit controls, unused filter controls, and no-template controls (NTC) along Year 7 samples. Only ASVs at or above 5% relative abundance are shown.

**References**

1. Usman MO, Simpson MJ. 2021. Assessment of the molecular-level compositional heterogeneity of natural organic matter in bentonites intended for long-term used nuclear fuel storage. Org Geochem 152:104166. <https://doi.org/10.1016/j.orggeochem.2020.104166>
2. Tong H, Behazin M, Simpson MJ. 2023a. Assessment of heat and radiation impacts on natural organic matter composition in bentonite for used nuclear fuel disposal. Appl Clay Sci 232:106808. <https://doi.org/10.1016/j.clay.2022.106808>
3. Tong H, Behazin M, Simpson MJ. 2023b. Assessment of salinity and heat impacts on natural organic matter composition in bentonite for used nuclear fuel storage. Appl Geochem 156:105746. <https://doi.org/10.1016/j.apgeochem.2023.105746>
4. Marshall MHM, McKelvie JR, Simpson AJ, Simpson MJ. 2015. Characterization of natural organic matter in bentonite clays for potential use in deep geological repositories for used nuclear fuel. Appl Geochem 54:43–53. <https://doi.org/10.1016/J.APGEOCHEM.2014.12.013>
5. Man M, Tong H, Srikanthan N, Usman MO, Tully CS, Noel JJ, Behazin M, Binns WJ, Keech PG, Simpson MJ. 2024. Analysis of natural organic matter chemistry in bentonite clay under compaction using different dry densities and duration. Appl Geochem 166:105985. <https://doi.org/10.1016/j.apgeochem.2024.105985>
